# Feasibility of patient-reported outcomes measurement information system (PROMIS) computerized adaptive tests in patients receiving physical therapy: A mixed-methods study

**DOI:** 10.1371/journal.pone.0362652

**Published:** 2026-11-13

**Authors:** Erik-Jan A. Haan, Annet Doomen, Harriët Wittink, Henri Kiers, Remco M. Arensman, Caroline B. Terwee

**Affiliations:** 1 Research group Clinical Decision Making in Movement Care, HU University of Applied Sciences Utrecht, Utrecht, The Netherlands; 2 Department of Epidemiology and Data Science, Amsterdam UMC, Vrije Universiteit Amsterdam, Amsterdam, The Netherlands; 3 Center for Physical Therapy Research and Innovation in Primary Care, Julius Health Care Centers, Utrecht, The Netherlands; 4 Association for Quality in Physical Therapy (SKF), Zwolle, The Netherlands; 5 Amsterdam Public Health Research Institute, Methodology, Amsterdam, The Netherlands; 6 Amsterdam Public Health Research Institute, Quality of Care, Amsterdam, The Netherlands; Northwestern University Feinberg School of Medicine, UNITED STATES OF AMERICA

## Abstract

**Introduction:**

Patient-Reported Outcome Measures (PROMs) are widely used in physical therapy, but both clinicians and patients report respondent burden due to the number and the length of existing instruments. PROMIS^®^ Computerized Adaptive Tests (CATs) may reduce this burden, yet their feasibility in primary care physical therapy is unknown. This study examined the feasibility of Dutch-Flemish PROMIS CATs in patients with musculoskeletal (MSK) disorders of the spine and upper extremity.

**Methods:**

We conducted a mixed-methods concurrent triangulation study in 14 physical therapy practices where PROMIS CATs were integrated into the electronic health record system and administered during routine care. A survey assessed physical therapists’ experiences and opinions regarding acceptability, demand, practicality, and implementation. Semi-structured interviews with physical therapists and patients further explored these domains. Survey data were analyzed descriptively, and interview transcripts were thematically analyzed by two independent researchers.

**Results:**

Twenty-five physical therapists completed the survey. Ten physical therapists and nine patients participated in interviews. Most physical therapists reported that PROMIS CATs were easy to use and facilitated focused discussions about symptoms and functioning. Patients described CATs as quick to complete and relevant to their condition and were willing to complete PROMs if results were used in consultations. Reported barriers included the simultaneous use of legacy PROMs (patients), difficulties using the online portal, and uncertainty about interpreting CAT scores (therapists). Some participants reported that certain CAT items felt less relevant and expressed a desire to view individual item responses.

**Conclusions:**

PROMIS CATs are considered feasible to implement in physical therapy practice and are generally experienced as acceptable, efficient, and clinically useful. Future implementation should focus on phasing out legacy PROMs to reduce respondent burden, improving skills in score interpretation, ensuring financial reimbursement for use, incorporating PROMIS into clinical guidelines, and evaluating additional PROMIS domains relevant to primary care physical therapy.

## Introduction

Patient-Reported Outcome Measures (PROMs) are increasingly used in primary care physical therapy in the Netherlands, to monitor symptoms, functioning, and treatment outcomes. However, both clinicians and patients frequently report high respondent and administrative burden due to the number of instruments in use, variability in content, and the time required to complete multi-item questionnaires [1,2]. These challenges can hinder routine use and limit the clinical value of PROMs, particularly in settings with high patient flow such as primary care physical therapy.

The Patient-Reported Outcomes Measurement Information System (PROMIS^®^) was developed to address these limitations of traditional PROMs by providing large, calibrated item banks based on item response theory [3,4]. PROMIS item banks can be administered as fixed short forms or Computerized Adaptive Tests (CATs). A CAT dynamically selects the most informative items from the underlying item bank based upon the respondent’s previous answer. Therewith, PROMIS CATs typically reduce the number of items administered while maintaining high measurement precision [5,6]. Because PROMIS domains are generic rather than disease-specific, they offer opportunities to harmonize outcome assessments across patient groups and clinical contexts [7].

PROMIS item banks for adults have been translated for Dutch-Flemish (DF) populations and extensively validated following international standards [8]. Within musculoskeletal (MSK) care and physical therapy settings in particular, several studies have demonstrated strong psychometric properties for relevant PROMIS domains. These include the DF Pain Interference item bank v1.1 (DF-PROMIS-PI v1.1) [9], the DF Physical Function item bank v1.2 (DF-PROMIS-PF v1.2) validated in patients receiving physical therapy [10], and the DF Upper Extremity (UE) item bank v2.0 (DF-PROMIS-UE v2.0) [11–13]. More recently, PROMIS CAT measurement properties in Dutch-Flemish adults have further been examined, including test-retest reliability, minimal detectable change (MDC), minimal important change (MIC), and responsiveness [14,15]. Together, these studies provide a solid psychometric foundation for using PROMIS CATs in clinical practice.

Despite these promising properties, the feasibility of PROMIS CATs in routine physical therapy care has not yet been evaluated. In this setting, time constraints, digital workflows, and the parallel use of legacy (traditional, well-established) PROMs may influence the usability and perceived value of PROMIS CATs [16,17]. Moreover, physical therapists (PTs) require clear graphical feedback (visual displays of PROM scores) and accessible score interpretation to integrate PROMs into clinical reasoning and shared decision-making [18]. Evidence from PROM implementation studies in hospital care suggests that CAT-generated feedback can support more focused consultations [19,20], but such findings cannot be assumed to generalize to the dynamic and heterogeneous workflows of primary care physical therapy.

Understanding the feasibility of PROMIS CATs in physical therapy is therefore an important next step toward broader and more efficient use of PROMIS CATs in this setting. Bowen’s feasibility framework provides a structured approach for early-stage implementation, focusing on acceptability, demand, practicality, and implementation [21]. Assessing these domains in a real-world setting, where PROMIS CATs are integrated into the electronic health record (EHR) system, and administered during routine care, can yield important insights into facilitators and barriers relevant for broader adoption.

The aim of this study was to evaluate the feasibility of DF-PROMIS CATs among patients with MSK conditions of the spine and UE receiving physical therapy. Using a mixed-methods design, we combined a survey among PTs working in private practice with in-depth interviews with therapists and patients to explore experiences, perceived value, and practical considerations related to the acceptability, demand, practicality, and implementation of DF-PROMIS CATs. This approach allowed us to assess PROMIS CATs under naturalistic conditions and to identify key factors that may facilitate or be a barrier to successful implementation in physical therapy practice.

## Methods

### Study design

We conducted a mixed-methods concurrent triangulation study [22] to evaluate the feasibility of Dutch-Flemish PROMIS^®^ Computerized Adaptive Tests (CATs) in primary care physical therapy. Quantitative data were collected through a survey of PTs, and qualitative data were obtained through semi-structured interviews with PTs and patients. Feasibility was assessed according to Bowen’s domains of acceptability, demand, practicality, and implementation which corresponds to the ‘can it work’-phase of intervention development (S1 Table).

### Ethics statement

This study is reported according to the consolidated criteria for reporting qualitative research (COREQ) 32-item checklist (S2 Table) [23].The study was reviewed by the Ethics Committee Research Healthcare Domain (ECO-GD) from the HU University of Applied Sciences which declared this research project not subject to the Dutch Medical Research Involving Human Subjects Act and that the protocol was in accordance with the Dutch Personal Data Protection Act (file number 167-000-2022). The study was conducted in accordance with the Declaration of Helsinki and all participants provided written informed consent.

### Setting and participants

The study was conducted across 14 primary care physical therapy practices in the Netherlands that participated in a cohort study between September 2021 and January 2023 [14,15]. All practices were affiliated with the Association for Quality in Physical Therapy (SKF) and used the EHR system Fysiomanager^®^ (Heerenveen, the Netherlands) into which DF-PROMIS CATs had been integrated for routine clinical use.

Eligible participants were: 1) PTs providing treatment for MSK conditions of the spine or UE, and 2) Adult patients (≥18 years) receiving treatment for MSK conditions. Exclusion criteria were: 1) not being able to complete questionnaires; or 2) insufficient command of the Dutch language. Based on clinical judgement, the treating PT assessed whether the patient could sufficiently understand the Dutch-language study information and questionnaires to participate. No standardized language proficiency assessment or predefined cut-off was used. This criterion was not included as an item in the questionnaire for PTs.

We aimed to interview approximately ten PTs and ten patients. This pragmatic target was expected to provide sufficient data richness and thematic coverage within the scope of this mixed-methods feasibility study. Purposive sampling was initially planned to obtain variation in participant characteristics; however, no formal selection criteria or quotas beyond the general eligibility criteria were predefined. In practice, PTs were recruited by convenience sampling based on their willingness and availability to participate [24]. Patients were informed about the study by their PT and were contacted by the research team only after providing permission. Limited willingness to participate, particularly among patients, also resulted in reliance on convenience sampling for the patient interviews. Recruitment resulted in interviews with ten PTs and nine patients.

### PROMIS CAT integration

The DF-PROMIS-PF v1.2, DF-PROMIS-UE v2.0, and DF-PROMIS-PI v1.1 CATs were embedded in the EHR-system via an Application Programming Interface (API) integration with the CAT software of the Dutch-Flemish PROMIS National Center. Each DF-PROMIS CAT begins with the most informative item for the average of the general population, and the patient’s response is used to estimate a T-score and its corresponding standard error (SE[T-score]). Subsequently, the most informative items are selected iteratively based on the updated estimate, and the process continues until a prespecified stopping rule is met. The stopping rules were either an SE(T-score) < 2.24, corresponding to a reliability of approximately 0.95, with a minimum of four completed items, or a maximum of 12 items. At the time of this study, the Dutch-Flemish PROMIS CATs used an SE(T-score) < 2.24 as the precision-based stopping rule, corresponding to a reliability of approximately 0.95 [14]. This relatively stringent threshold was selected for clinical use in the Netherlands to obtain highly precise scores at the individual-patient level. A minimum of four items was administered, and the CAT stopped after a maximum of twelve items if the precision criterion had not been reached.

Final scores are expressed as standardized T-scores (mean 50, SD 10) referenced to the US general population. Visual feedback for each PROMIS domain was integrated into the EHR-system as recommended by the Dutch-Flemish PROMIS National Center. Each domain was displayed in a separate longitudinal graph, in which numerical T-scores were presented as dots connected by a blue line. The graph was overlaid on a traffic-light heat map indicating reference cut-off ranges, with orange representing moderate and red representing severe scores. A visual example and further explanation of this graphical display are provided by Van Muilekom et al. [25].

CATs were completed via a patient portal at home or during consultations using the PTs’ personal computer. PTs received a short introduction and a written reference guide explaining CAT administration, interpretation of T-scores, and viewing visual feedback.

Because use of PROMIS CATs involves a service fee (approximately €0.15 excl. VAT per CAT administration), these costs were fully covered by the research project. Participating practices therefore did not incur any expenses for CAT use during the study period.

CAT administration occurred alongside the use of legacy PROMs as part of routine care. Legacy PROMs, as mandated by national quality registries, were the Numerical Pain Rating Scale (NPRS) [26] and Patient-Specific Functional Scale (PSFS) [27] for all conditions, Quebec Back Pain Disability Scale (QBPDS) for low back pain [28], Neck Disability Index (NDI) for neck pain [29], and Quick-Disabilities of the Arm, Shoulder and Hand (QuickDASH) questionnaire for UE conditions [30]. The order of administration was not standardized, as each practice integrated the PROMIS CATs into its own EHR–based data collection workflow. Consequently, the order of PROMIS CATs and legacy PROMs may have varied across practices and participants.

### Data survey

An online survey (completed between May 2 and June 20, 2022) assessed PTs’ experiences with PROMIS CATs across Bowen’s feasibility domains [21]. The survey contained Likert-scale items, multiple-response questions, and optional free-text fields. Survey construction was informed by existing PROM implementation literature [19,31] and Bowen’s framework. Descriptive statistics (frequencies, percentages, means where appropriate) were calculated for all closed-ended items; free-text responses were summarized in order of frequency. The full survey is provided in S3 Table. A secured data collection tool (Crowdtech^®^) was used and reminders were sent after one week and again after two weeks. Participants provided informed consent in a check box in the digital survey.

### Interviews

#### Interviewers.

Interviews were conducted by the first author (EH) and trained PT interviewers (AJ, EM, MK, JO, HA). Their backgrounds included manual therapy, sports PT, mental health PT, and pediatric PT. All interviews were conducted with two interviewers present: one lead interviewer and one observer/assistant who asked supplementary questions and took field notes.

### Interview procedures

Topic guides (S4 Table) for PTs and patients were developed based on Bowen’s domains, prior PROM implementation studies [19,31], and the research team’s clinical experience.

The topic guides were piloted in three test interviews to assess question clarity, interview flow, and the distinction between PROMIS CATs and legacy PROMs. No major changes were made to the overall content or feasibility domains. Based on the pilot interviews, the order and wording of several questions were refined, and interviewers were instructed to show participants an example of a PROMIS CAT with its graphical feedback alongside an example of a legacy PROM. Additional prompts were added to clarify which instruments participants had completed, whether completion occurred at home or during the consultation, and whether participants wished to view individual item responses. The pilots were also used to train interviewers in asking open, non-leading questions, using follow-up probes and summaries, allowing pauses, and conducting the interview as a conversation rather than as a fixed sequence of questions. Pilot interviews were not included in the final analysis.

At the end of each interview, participant characteristics were collected. Education level and digital skills were self-reported using pragmatic categories (education: high, middle, or low; digital skills: high, average, or low); no standardized definitions or classification instruments were used. Interviews were conducted between March 21 and November 23, 2022, via secure MS Teams due to COVID restrictions, audio-recorded, and lasted 32–62 minutes.

### Transcription and data management

All interviews were transcribed verbatim. Twelve interviews were transcribed by PT interviewers (EM, MK, HA, JO) with support from Amberscript automatic transcription software (www.amberscript.com). Seven interviews were transcribed by a professional transcription service (www.e-kern.com). The first author (EH) checked all transcripts for accuracy.

### Qualitative analysis

Qualitative data were analyzed using a hybrid inductive–deductive thematic analysis [32,33]. Two researchers (EH and AJ or AD) independently performed open coding of each initial transcript. The two coders compared their codes during consensus meetings and resolved differences through discussion. When consensus could not be reached, HW reviewed the relevant transcript excerpt and codes and made the final decision. Coding disagreements were documented in a separate disagreement log.

Codes were subsequently grouped into themes derived inductively from the data. Themes were mapped deductively onto Bowen’s feasibility domains. We did not set a priori saturation criteria: in line with recent recommendations for mixed-methods and feasibility research, we focused instead on data richness and thematic coverage [34,35]. Coding and theme development were facilitated using Atlas.ti computer software (www.Atlasti.com). Analytic rigor was strengthened through regular peer debriefing sessions within the research team (EH, AD, HW). Member checking was offered by email; no participants requested changes.

### Mixed-methods integration

Quantitative and qualitative data were initially analyzed separately. Following completion of both analyses, the first and second author (EH and AD) compared the survey results and interview themes within each of Bowen’s feasibility domains: acceptability, demand, practicality, and implementation. Findings were considered convergent when the quantitative and qualitative results pointed in the same direction, complementary when the interview findings provided further explanation or context for the survey results, and divergent when findings appeared inconsistent or reflected contrasting experiences. Potential differences were discussed, taking into account that the survey included PTs only, whereas the interviews included both PTs and patients. Agreement and disagreement were evaluated qualitatively rather than by using a formal concordance score. This comparison informed the integrated summary presented for each feasibility domain in the Results section.

## Results

### Participants

Twenty-five PTs, 14 (56%) females and 11 (44%) males, completed the survey. Fifteen therapists were specialized in manual therapy, two in sports physical therapy, one in mental health physical therapy, five were hand therapists, and five had no specialty. The mean age was 39.4 (range 27–63, SD 11.5) years, with average working hours per week of 29.4 (SD 6.8) and working experience of 16.6 (SD 11.1) years. Ten PTs and nine patients were interviewed. Interviewed PTs varied in age, years of work experience, and clinical focus. Interviewed patients varied in age, symptom location, education level and digital skills (Tables 1 and 2). Interviews lasted 32–62 (mean 47, SD 8) minutes.

**Table 1:** Characteristics of interviewed physical therapists.

Participant ID	Interview duration (min)	Age (years)	Sex	Working experience (years)	Specialty
T01	51.7	27	Female	2	General physical therapist
T02	51.7	28	Female	7	Hand therapist
T03	42.6	39	Female	17	Manual therapist
T04	62.3	36	Female	10	General physical therapist
T05	49.5	37	Female	15	General physical therapist
T06	50.3	28	Female	7	Manual therapist
T07	56.8	37	Male	17	Manual therapist
T08	42.3	35	Female	14	Manual therapist
T09	52.0	29	Female	7	Hand therapist
T10	46.6	70	Male	45	Manual therapist

**Table 2:** Characteristics of interviewed patients.

Participant ID	Interview duration (min)	Age (years)	Sex	Main complaint	Self-reported education level	Self-reported digital skills
P01	33.2	38	Female	Shoulder	High	Average
P02	58.9	48	Male	Back	High	High
P03	57.0	28	Male	Back	High	High
P04	37.6	37	Female	Neck	High	High
P05	44.4	32	Female	Back	High	High
P06	43.7	22	Female	Back	Middle	High
P07	34.9	44	Male	Neck	High	High
P08	37.9	58	Male	Elbow	High	High
P09	40.0	67	Male	Back	Low	Average

### Survey findings

Responses to the survey are presented in Table 3 and Table 4, Table 5, Table 6, Table 7, Table 8.

**Table 3:** Responses to survey questions using a 5-point Likert scale.

Item		Completely agree		Agree		Don’t agree / don’t disagree		Disagree		Completely disagree		% Agreeing
		N	(%)	N	(%)	N	(%)	N	(%)	N	(%)	
	**Acceptability**											
6.	Using PROMIS CATs does not affect my professional authority to make my own decisions.	6	(24.0)	13	(52.0)	5	(20.0)	1	(4.00)	0	(0.0)	**76.0**
7.	I find that PROMIS CAT scores helps me to formulate a physical therapy diagnosis.	1	(4.0)	10	(40.0)	8	(32.00)	5	(20.0)	1	(4.0)	44.0
8.	I find that a PROMIS CAT score contributes to the evaluation of physical therapy treatment.	5	(20.0)	14	(56.0)	3	(12.0)	2	(8.0)	1	(4.0)	**76.0**
10.	I find that the use of PROMIS CATs contributes to the quality of physical therapy healthcare.	4	(16.0)	14	(56.0)	4	(16.0)	2	(8.0)	1	(4.0)	**72.0**
15.	When using PROMIS CATs I am able to include my patients’ wishes.	4	(16.0)	15	(60.0)	5	(20.0)	1	(4.0)	0	(0.0)	**76.0**
16.	I have a positive attitude towards the use of PROMIS CATs.	6	(24.0)	11	(44.0)	5	(20.0)	1	(4.0)	1	(4.0)	**68.0**
19.	I have experienced the added value of using PROMIS CATs.	2	(8.0)	6	(24.0)	9	(36.0)	7	(28.0)	1	(4.0)	32.0
21.	Using PROMIS CATs is a standard part of my clinical reasoning.	2	(8.0)	0	(0.0)	11	(44.0)	10	(40.0)	2	(8.0)	8.0
37.	I think patients are satisfied with the use of PROMIS CATs.	0	(0.0)	6	(24.0)	14	(56.0)	5	(20.0)	0	(0.0)	24.0
41.	The graphs of the PROMIS CAT scores have an attractive lay-out.	2	(8.0)	9	(36.0)	12	(48.0)	2	(8.0)	0	(0.0)	44.0
42.	I am satisfied with the PROMIS CAT Physical Functioning.^a^ (n=22)	1	(4.5)	10	(45.5)	7	(31.8)	3	(13.6)	1	(4.5)	50.0
43.	I am satisfied with the PROMIS CAT Upper Extremity.^b^ (n=22)	2	(9.1)	8	(36.4)	8	(36.4)	3	(13.6)	1	(4.5)	45.5
44.	I am satisfied with the PROMIS CAT Pain Interference.^c^ (n=24)	2	(8.3)	12	(50.0)	8	(33.3)	2	(8.3)	0	(0.0)	**58.3**
45.	I am satisfied with the graphical feedback (display of scores over time and comparison with the T-score of 50 as an average for the general population).	0	(0.0)	11	(44.0)	13	(52.0)	1	(4.0)	0	(0.0)	44.0
46.	I am satisfied with the cut-off lines and labels in the graph (mild, moderate, severe).	1	(4.0)	12	(48.0)	12	(48.0)	0	(0.0)	0	(0.0)	**52.0**
47.	I would also like to see back the scores on the separate questions (items) of the CAT.	4	(16.0)	10	(40.0)	5	(20.0	4	(16.0)	2	(8.0)	**56.0**
52.	Within our practice, we have agreed on how to use PROMIS CATs.	4	(16.0)	16	(64.0)	4	(16.0)	1	(4.0)	0	(0.0)	**80.0**
58.	The use of PROMIS CATs fits well in the way of working in my practice/institution.	5	(20.0)	13	(52.0)	4	(16.0)	3	(12.0)	0	(0.0)	**72.0**
59.	The use of PROMIS CATs is included in the policy of our practice/institution.	1	(4.0)	6	(24.0)	7	(28.0)	8	(32.0)	3	(12.0)	28.0
	**Demand**											
2.	I am interested in using PROMIS CATs.	8	(32.0)	12	(48.0)	3	(12.0)	2	(8.0)	0	(0.0)	**80.0**
11.	I find it important to objectively capture patient data through PROMIS CATs.	4	(16.0)	13	(52.0)	7	(28.0)	1	(4.0)	0	(0.0)	**68.0**
12.	I believe that the use of PROMIS CATs should be a part of physical therapy clinical reasoning.	4	(16.0)	6	(24.0)	12	(48.0)	2	(8.0)	1	(4.0)	40.0
17.	I would like to use PROMIS CATs more often in my practice.	5	(20.0)	9	(36.0)	7	(28.0)	2	(8.0)	2	(8.0)	**56.0**
18.	I use PROMIS CATs in daily practice.	2	(8.0)	5	(20.0)	9	(36.0)	7	(28.0)	2	(8.0)	28.0
	**Practicality**											
3.	I know where to find PROMIS CATs in my Electronic Health Record (EHR).	18	(72.0)	6	(24.0)	1	(4.0)	0	(0.0)	0	(0.0)	**96.0**
4.	I am able to administer PROMIS CATs to my patients.	16	(64.0)	9	(36.0)	0	(0.0)	0	(0.0)	0	(0.0)	**100.0**
5.	I am able to correctly interpret PROMIS CAT scores.	7	(28.0)	10	(40.0)	7	(28.0)	1	(4.0)	0	(0.0)	**68.0**
9.	I know how to apply PROMIS CATs within my clinical reasoning process.	4	(16.0)	18	(72.0)	3	(12.0)	0	(0.0)	0	(0.0)	**88.0**
13.	I find that working with PROMIS CATs takes little time.	5	(20.0)	5	(20.0)	10	(40.0)	3	(12.0)	2	(8.0)	40.0
14.	Patients find that using PROMIS CATs takes little time.	1	(4.0)	5	(20.0)	13	(52.0)	4	(16.0)	2	(8.0)	24.0
20.	Patients are cooperative in using PROMIS CATs.	1	(4.0)	11	(44.0)	9	(36.0)	4	(16.0)	0	(0.0)	48.0
22.	I have sufficient routine in the use of PROMIS CATs.	4	(16.0)	8	(32.0)	7	(28.0)	6	(24.0)	0	(0.0)	48.0
28.	All patients are invited to complete the PROMIS CATs online.	3	(12.0)	4	(16.0)	6	(24.0)	5	(20.0)	5	(20.0)	28.0
31.	I find that PROMIS CAT can easily be administered during the consultation.	1	(4.0)	9	(36.0)	7	(28.0)	6	(24.0)	2	(8.0)	40.0
33.	When I use PROMIS CATs, I identify patient problems faster.	0	(0.0)	2	(8.0)	13	(52.0)	9	(36.0)	1	(4.0)	8.0
40.	PROMIS CATs are easy to use.	1	(4.0)	17	(68.0)	4	(16.0)	3	(12.0)	0	(0.0)	**72.0**
49.	I know where to go with my questions about PROMIS and PROMIS CAT	7	(28.0)	16	(64.0)	1	(4.0)	1	(4.0)	0	(0.0)	**92.0**
50.	I need training on PROMIS and PROMIS CAT	0	(0.0)	7	(28.0)	7	(28.0)	9	(36.0)	2	(8.0)	28.0
61.	I always have PROMIS CATs completed by the patients themselves.	9	(36.0)	9	(36.0)	4	(16.0)	3	(12.0)	0	(0.0)	**72.0**
	**Implementation**											
53.	Management supports staff in the use of PROMIS CATs.	5	(20.0)	10	(40.0)	9	(36.0)	1	(4.0)	0	(0.0)	**60.0**
54.	Management itself uses PROMIS CATs.	3	(12.0)	12	(48.0)	10	(40.0)	0	(0.0)	0	(0.0)	**60.0**
55.	Management requires staff to use PROMIS CATs.	2	(8.0)	4	(16.0)	4	(16.0)	11	(44.0)	4	(16.0)	24.0
56.	Fellow therapists in my practice/institution use PROMIS CATs.	3	(12.0)	11	(44.0)	8	(32.0)	3	(12.0)	0	(0.0)	**56.0**
57.	In our practice/institution, we discuss how to implement PROMIS CATs.	1	(4.0)	10	(40.0)	8	(32.0)	3	(12.0)	3	(12.0)	44.0

^a^Three respondents (12%) indicated “not applicable/did not use”; percentages are calculated on n = 22.

^b^Three respondents (12%) indicated “not applicable/did not use”; percentages are calculated on n = 22.

^c^One respondent (4%) indicated “not applicable/did not use”; percentages are calculated on n = 24.

**Table 4:** Survey items with categorical response options (N = 25).

Item	Question	Response options	Distribution (N, %)
23.	I discuss the PROMIS CAT scores with patients.	Always / Mostly / Sometimes / Almost never / Never	6 (24) / 6 (24) / 7 (28) / 5 (20) / 1 (4)
25.	I discuss the PROMIS CAT scores at the start/ middle/end of the treatment trajectory.	Start / Middle / End	18 (72) / 12 (48) / 16 (64)
27.	To what extent are you satisfied with the time spent discussing PROMIS CAT scores?	Satisfied / More time / Less time	17 (68) / 2 (8) / 6 (24)
30.	I estimate that …% of patients complete the PROMIS CATs online.	0 / 0–25 / 25–50 / 50–75 / 75–100	2 / 9 / 4 / 5 / 5
63.	I use PROMIS CATs in …% of my patients with musculoskeletal complaints.	0 / 1–10 / 11–20 / 21–50 / 51–80 / 81–100	0 / 12 / 7 / 4 / 1 / 1

**Table 5:** Open ended reasons for limited discussion or completion.

Item	Question	Open text response (times mentioned)
24.	The reason that I do not always discuss the PROMIS CAT scores with patients is. (n = 22)	• Insufficient time (6) • not always relevant or not informative items (3) • I don’t know how to interpret/ CAT measurement properties still under research (3) • not always a follow up session planned or no-show by patient (3) • I use/discuss other PROM scores (2) • not necessary (1) • patient is not interested (1) • scores not in my dashboard/ treatment plan (1) • patient gives own interpretation to scores (1) • forgot (1).
29.	The reason not all patients are invited to complete the PROMIS CAT online are. (n = 17)	• lack of time (5) • patient not included for research project (5) • logistic/administrative issue or no email (4) • I use other PROMs (2) • privacy (1).
32.	The reason a PROMIS CAT cannot easily be administered during the consultation is. (n = 14)	• not enough time (7) • other PROMs should also be administered/too many PROMs (3) • online is more efficiently (1) • computer not available (1) • no relevant items which confuse the patient (1) • possibility for the therapist to influence how the patient scores items (1).
34.	Reasons why I don’t identify patient problems faster, when I use PROMIS CATs (n = 18)	• history taking/clinical reasoning is more informative/specific (7) • no benefit of CAT compared to other PROMs (4) • psycho-social items are not covered in the (PF or PI) CAT (2) • unable to see the individual item scores of the CAT (2) • CAT does not help me as a diagnostic instrument (1) • experience as a therapist (1) • don’t know (1).

**Table 6:** Benefits and disadvantages.

Item	Question	Open text response (times mentioned)
35.	Benefits of PROMIS CATs are (multiple answers possible)	• short completion time compared to legacy PROMs (11) • adaptive so more patient-centered PROM (4) • more generic measure/not disease specific (3) • don’t know/no opinion (2) • (hopefully) less PROMs needed in the future (2) • easy for goal setting and monitoring outcomes (2) • precise measurement (1) • standardization (1) • external accountability to health insurers (1)
36.	Disadvantages of PROMIS CATs are (multiple answers possible) (n = 21)	• time needed to administer (7) • additional to legacy PROMs/too many PROMs (3) • individual item scores cannot be seen (3) • not always relevant items or duplicate items (3) • patients do not always complete CATs online (3) • not suitable for every patient (1) • if mandatory than not always applicable in daily practice (1)
38.	Benefits for patients according to therapist to use PROMIS CATs are (multiple answers possible)	• short/fast/less time needed to administer/less items (12) • better insight in functioning/ progress (4) • no opinion/don’t know (4) • easy to complete (online/ in advance) (3) • compare with normative values (1) • only one PROM necessary (1)
39.	The most frequently heard comments from patients about PROMIS CATs are (n = 18)	• to many questionnaires/yet another questionnaire? (9) • none (4) • sometimes irrelevant items/not applicable or duplicate items (3) • easy/less time to complete than expected (2)

**Table 7:** Preferred additional PROMIS domains (N = 25).

Domain	N	(%)
Anxiety	10	40
Depression	8	32
Fatigue	6	24
Pain Behavior	8	32
Physical Function – Mobility (lower extremity)	12	48
Sleep Disturbance	6	24
Sleep-Related Impairment	4	16
Ability to Participate in Social Roles and Activities	7	28
Satisfaction with Social Roles and Activities	1	4
Self-Efficacy for Managing Chronic Conditions – Manage Meds/Treatment	3	12
Self-Efficacy for Managing Chronic Conditions – Manage Symptoms	8	32

**Table 8:** Training, policy, facilitators and barriers.

Item	Question	Open text response (times mentioned)
51.	I would like to see the following topics covered in training. (multiple answers possible)	• not applicable/don’t know/no answer (12) • interpretation of scores (8) • how to use/implement in clinical practice (5) • measurement properties (1) • usability (1)
60.	What is the reason that the use of PROMIS CATs in not included in the policy of your practice? (n = 16)	• project is still in research phase (8) • not applicable/don’t know/no answer (4) • no added value compared to other measures (2) • not enough experience/new (2).
62.	For what reason did you or someone else help the patient to complete the PROMIS CAT? (n = 7)	• patient did not/could not complete (online) (5) • lack of time (1) • patient forgot reading glasses (1)
64.	What are facilitators for the use of PROMIS CATs?	• Short/fast/time to complete (7) • no opinion/no answer/don’t know (7) • easy to interpret/insight in functioning/ unambiguity (4) • can replace other PROMs like PSFS and NPRS (2) • standardization in procedure (1) • obligatory use for quality registry (1) • automation/online (1) • better knowledge of interpretation (1) • good staff support (1)
65.	What are barriers for the use of PROMIS CATs?	• too many PROMs mandatory for quality registry and/or for this research project (6) • no answer/don’t know (5) • patient does not complete CAT/PROMs online (4) • time (e.g., compared to NPRS) (3) • prefer the PSFS/cant’s use CAT for goal setting and evaluation (2) • usability of completion during consultation (1) • cannot see individual items (1) • design of the patient tracking system (1) • lack of experience (1) • forget to complete/reserve time during consultation (1)

Survey and interview findings are presented per Bowen’s domain. Survey results are completed by qualitative themes derived from the interviews, which are arranged according to the same domains. Themes and supporting quotes are presented in Table 9.

**Table 9:** Positive experiences and challenges/suggestions per theme mentioned by PTs and patients in the interviews.

Domain/theme	Quote	
	*Positive experiences*	*Challenges/suggestions*
Acceptability: **Clinical reasoning** **and evaluation**	*PT#2: “The graph helps patients see their progress, even when symptoms are still there.”* *Patient#5: “Answering the questions made me reflect on my symptoms and what I want help with.”*	*PT#3: “I treat mobility impairments the same regardless of the score; I can’t always link the questionnaire results directly to my treatment choices.”*
Acceptability: **Graphical feedback** **and interpretation**	*PT#2: “I didn’t look at the numbers; the heatmap colors and labels like mild or severe were clear for me.”* *Patient#3: “If the therapist explains the graph, you can really see progress even when complaints are still there; that makes it meaningful.”*	*PT#7: “Using words like ‘severe’ might unintentionally worry patients or make the problem feel bigger.”* *PT#6: “If patients see the scores without explanation, they may misinterpret the numbers and develop unhelpful thoughts.”*
Acceptability: **Adaptive mechanism and item content of PROMIS CAT**	*PT#8: “The adaptive questions are useful; with one CAT you may replace several longer questionnaires, which saves time.”* *Patient#4: “It felt like a good questionnaire because it zoomed in on what mattered to me, instead of asking irrelevant things.”*	*PT#10: [about the UE CAT] “Patients sometimes get irritated when questions feel repetitive; they think it’s the same question just worded differently.”* *Patient#8: [about the UE CAT] “Some questions didn’t match my situation; I had difficulties with several movements but none that I couldn’t do at all.”*
Demand: **Interest and willingness to use PROMs in general**	*Patient#6: “It helps you prepare for treatment, thinking about how severe your complaints are. And if it helps the physio help me better, then it’s only positive; you do it together.”* *PT#6: “Sometimes the CAT reveals something the patient didn’t mention in the history-taking; that can help clarify symptoms or even guide a different subgoal.”*	*PT#8: Only about 10–20% complete the questionnaires beforehand; people get questionnaire fatigue because we send many forms.”* *Patient#4: “You need to explain why the questionnaire matters and how it helps both sides.”*
Demand: **PROMIS CAT vs** **legacy PROMs**	*PT#10: “Those legacy questionnaires are far too long. If digital technology can streamline them and target only relevant questions, that would be fantastic.”* *PT#6: “It would be great if PROMIS CAT could replace both the PSFS and the Quebec; then you’d have one questionnaire instead of several.”* *PT#6: “If the CAT can replace NPRS, PSFS, and the Quebec while giving the same information, that’s a win-win.”* *PT#1: “I mainly use the CAT score for evaluation; interpreting and explaining it to the patient also becomes part of treatment.”*	*PT#4: “I don’t rely solely on the CAT; I still ask for NPRS and PSFS as well.”* *PT#1: “I mainly use the score for evaluation, but it doesn’t guide my treatment as much by itself.”* *Patient#3: “With the old questionnaire, you know what to expect, you can see upfront how many questions there are.”*
Practicality: **Ease of use**	*PT#1: It’s very easy to use; quick, accessible, and the program works smoothly.”* *Patient#7: “It stays clear because you only get one question at a time; that works well for me and probably for others too.”*	*PT#8: “It’s inconvenient that you have to click in the top corner to continue; it slows things down compared to automatic progression.”*
Practicality: **Digital skills and** **literacy barriers**	*Patient#8: “It doesn’t cost me any effort at all to do these things on the computer.”*	*PT#6: “Low literacy or language barriers can be a problem; reading aloud or translating changes the meaning of answers.”*
Practicality: **Devices and** **online portal**	*Patient#2: “It’s very simple; if you can use a browser and send an email, that’s enough.”*	*PT#4: “The online portal should work better; if patients can’t fill it out beforehand, they get frustrated when they must do it at the first visit.”* *PT#6: “People who are less computer-savvy find the portal complicated and then think: well, that questionnaire, never mind.”*
Implementation: **Knowledge and** **education**	*PT#1“It’s good that test–retest reliability [and other measurement properties] is being studied; we need to know how to interpret the scores and when to use which CAT.”.*	*PT#6: “It still feels unfamiliar; I didn’t really use it because I rely on NPRS and PSFS, so some training would help.”* *PT#7: “Training needs differ, some prefer a webinar they can watch anytime; others really need in-person explanation.”*
Implementation: **Organizational embedding**	*PT#9: “We work closely with the hospital, so it would be useful to see pre- and postoperative CAT results to understand how patients return to us.”* *PT#6: “If it clearly saves time for my therapists, then I would consider paying for the CATs.”*	*PT#8: “It’s a lot on top of everything we already do; with all the other required clinimetrics, it becomes quite a hassle.”* PT*#7: “If you want to implement something like this, it must be supported by the profession and included in guidelines. If it’s not widely backed and doesn’t replace existing* PROM*s, implementation will be difficult.”*
Implementation: **User driven improvements**		*PT#5: “When the CAT is completed online beforehand, you miss discussing the patient’s answers to individual items together.”* *Patient#9: “A graph is fine, but seeing the individual questions would show more clearly what someone can and cannot do.”* *Patient#3: “It would help to view a full overview before sending it, so you can correct something if you notice a mistake.”*

### Acceptability

#### Survey findings.

PTs generally considered PROMIS CATs acceptable and useful. Most agreed that CATs contribute to treatment evaluation (76%) and quality of care (72%). Satisfaction with the graphical feedback was moderate (44–52%), and only 24% agreed that patients were satisfied with using CATs. More than half (56%) wished to view individual item responses. Although 80% reported they agreed on how to use PROMIS CAT within their practice, structural policy embedding was limited (28%). CAT results were not always discussed with patients: 48% reported doing so always or mostly (Table 4). Reasons for not discussing scores included lack of time, perceived limited relevance of specific items, and uncertainty about interpretation of the scores (Table 5). PTs reported that CATs provide insight into patient functioning and were mainly experienced as short by patients (Table 6).

#### Interview findings.

Themes summarizing PT’s and patients’ views on acceptability were: 1) clinical reasoning and evaluation; 2) graphical feedback and interpretation; and 3) the adaptive mechanism and item content of PROMIS CAT.

### Clinical reasoning and evaluation

Both PTs and patients viewed PROMIS CATs as acceptable tools that support monitoring and evaluation of treatment. Patients appreciated completing PROMs in general because it helped them reflect on symptoms and goals; one patient noted, *“Answering the questions made me reflect on my symptoms and what I want help with”* (Patient#5). Contrary to PTs’ expectations, patients did not express resistance to completing PROMs. PTs primarily used CATs for monitoring change, especially in longer trajectories. Visual displays helped contextualize residual symptoms: *“The graph helps patients see their progress, even when symptoms are still there”* (PT#2). Some therapists, however, had difficulty linking PROMIS CAT (domain) scores directly to treatment decisions, as they were accustomed to using the PSFS and NPRS to guide their clinical reasoning. As one therapist explained: *“I can’t always link the questionnaire scores directly to my treatment choices”* (PT#3).

### Graphical feedback and interpretation

Many PTs and patients appreciated the graphical feedback, including longitudinal trends and the color-coded heatmap. Several described the visualization as motivating. Yet some PTs hesitated to share graphs because red color coding or labels such as “severe” might provoke unnecessary concern: *“Using words like ‘severe’ might unintentionally worry patients or make the problem feel bigger”* (PT#7). Other PTs questioned whether patients fully understood T-scores and cut-offs, or whether the comparison with a T = 50 norm score was meaningful from the patient perspective. As one PT expressed, *“If patients see the scores without explanation, they may misinterpret the numbers and develop unhelpful thoughts”* (PT#6).

### Adaptive mechanism and item content of PROMIS CAT

Both patients and PTs described PROMIS CATs as quick, simple, and more focused than conventional PROMs. One PT stated, *“The adaptive questions are useful; with one CAT you may replace several longer questionnaires”* (PT#8). A patient noted, *“It zoomed in on what mattered to me, instead of asking irrelevant things”* (Patient#4). However, some found CAT items repetitive or insufficiently specific, particularly in the Upper Extremity CAT. Although some interviewed patients reported confusion or impatience when CATs administered more items, patients generally described the CATs as quick and relevant. This contrasted with the survey finding that only 24% of PTs perceived patients to be satisfied with PROMIS CATs (Table 3, item 37) and only 24% thought that patients considered CAT completion time-efficient (Table 3, item 14).

### Integrated summary

Quantitative and qualitative findings converged in showing that PROMIS CATs were generally considered acceptable and useful for monitoring and evaluating treatment. The interview findings complemented the survey results by showing that graphical feedback could support reflection on progress, while concerns about the interpretation of T-scores, cut-offs, and color coding may explain the moderate satisfaction with graphical feedback and why CAT scores were not consistently discussed with patients. Both data sources also identified the perceived irrelevance of some items, particularly in the Upper Extremity CAT, as a barrier to acceptability. A divergence was observed regarding patient satisfaction: only 24% of PTs indicated in the survey that patients were satisfied with PROMIS CATs, whereas the interviewed patients generally described CATs as quick, simple, and relevant and did not express resistance to completing them. This difference may reflect the distinction between PTs’ perceptions of patient experiences and patients’ own accounts.

### Demand

#### Survey findings.

Most PTs expressed interest in using PROMIS CATs (80%) and considered objective monitoring important (68%), although only 28% reported regular use in daily practice. More than half (56%) wished to increase their usage. Open responses indicated that CATs were valued for their brevity, potential to replace other (legacy) PROMs, and standardization. However, administering PROMIS CATs in addition to several mandatory legacy PROMs within the Dutch quality registry context increased overall respondent burden, which may partly explain why expressed interest did not translate into routine use.

#### Interview findings.

Demand-related themes were: 1) interest and willingness (to use PROMs in general); and 2) using PROMIS CAT vs legacy PROMs.

### Interest and willingness to use PROMs

In general, most patients were motivated to complete PROMs (in general) when the purpose was clear, and results were discussed with them. PROMs helped them prepare for consultations and articulate symptoms; as one patient described, *“It helps you prepare, thinking about how severe your complaints are… you do it together”* (Patient#6). PTs acknowledged this value but highlighted the burden of administering multiple PROMs. They strongly preferred PROMIS CAT over legacy PROMs, but not in addition to them. One PT explained that questionnaire overload reduced online completion by patients: *“Only about 10–20% fill them out beforehand; people get questionnaire fatigue”* (PT#8).

### PROMIS CAT vs legacy PROMs

PTs appreciated the adaptive and personalized nature of CATs. Some saw clear potential for replacement: *“If the CAT can replace NPRS, PSFS, and Quebec while giving the same information, that’s a win-win”* (PT#6). Patients appreciated CAT relevance but sometimes preferred seeing all items at once, as with fixed-form PROMs. Many PTs felt CATs were useful for general monitoring but less suited to specific goal setting. Some PTs continued using familiar tools such as NPRS and PSFS: *“I don’t rely solely on the CAT; I still ask for NPRS and PSFS as well”* (PT#4).

### Integrated summary

Survey and interview findings converged in showing considerable interest in PROMIS CATs, but limited routine use. The interviews complemented the survey findings by showing that patients’ willingness to complete PROMs depended on a clear purpose and discussion of the results, while PTs preferred PROMIS CATs as a replacement for, rather than an addition to, legacy PROMs. The simultaneous use of mandatory legacy PROMs and PTs’ continued reliance on familiar instruments for specific goal setting help explain why the expressed demand for PROMIS CATs did not translate into routine use.

### Practicality

#### Survey findings.

Most PTs found PROMIS CATs easy to locate in the EHR-system (96%) and to administer (100%). Most PTs rated CATs as easy to use (72%), and 88% knew how to incorporate them into clinical decision-making. Time remained a concern: only 40% felt CATs took little time, and 24% believed patients found them time efficient. Logistical issues for online PROM collection (PROMs in general) such as difficulties with the patient portal, missing email addresses, and incomplete online PROMs were noted although not specifically for CAT completion (Table 5).

#### Interview findings.

Practicality-related themes were: 1) ease of use; 2) digital skills and literacy barriers; and 3) devices and online portal.

### Ease of use

Most PTs and patients found CATs straightforward and quick. One PT said, *“It’s very easy to use; quick, accessible, and the program works smoothly”* (PT#1). Patients appreciated the simple, one-item-at-a-time layout: *“It stays nice and clear; you only get one question at a time”* (Patient#7). A few PTs noted minor interface frustrations for CAT completion (e.g., needing to click “continue” in an inconvenient screen location).

### Digital skills and literacy barriers

All interviewed participants self-reported they had adequate digital skills. Still, PTs anticipated challenges among patients with low literacy or limited Dutch proficiency. As one PT put it, *“Low literacy or language barriers can be a problem: reading aloud or translating changes the meaning of answers”* (PT#6).

### Devices and the online portal

Patients generally had access to devices and described online completion as simple; *“If you can use a browser and send an email, that’s enough”* (Patient#2). PTs, however, frequently encountered login issues, forgotten passwords, or incomplete online PROMs. One PT noted, *“If patients have to fill it out during the first visit, they get frustrated, and so do I”* (PT#4). These challenges reflected broader issues associated with online PROM administration and were not specific to the adaptive PROMIS CATs.

### Integrated summary

Survey and interview findings converged in showing that PROMIS CATs themselves were easy to access, administer, and use. An apparent divergence concerned time efficiency: interviewed PTs and patients generally described CAT completion as quick, whereas only 40% of surveyed PTs considered CATs time-efficient and 24% thought patients did so. The interview findings suggest that this difference mainly reflected broader workflow barriers related to the online portal and PROM administration rather than the adaptive CAT itself.

### Implementation

#### Survey findings.

Management support for PROMIS CATs was moderate (60%), and mandatory use was uncommon (24%). More than half of PTs reported that colleagues used CATs, but fewer (44%) indicated that implementation was discussed within their practice. PTs expressed the need for more education, especially on score interpretation and using CATs for clinical reasoning.

#### Interview findings.

Implementation-related themes were: 1) knowledge and education; 2) organizational embedding and 3) user drive improvements.

### Knowledge and education

In general, PTs felt confident interpreting CAT scores and applying the results within their clinical reasoning. However, many requested trainings on T-scores, cut-offs, MIC/MDC, and practical use. One PT summarized: *“We need to know how to interpret the scores and when to use which CAT”* (PT#1). Others described unfamiliarity as a barrier: *“It still feels unfamiliar… I rely on NPRS and PSFS, so some training would help”* (PT#6). Most PTs preferred short webinars, e-learning modules, or practical handouts.

### Organizational embedding

Therapists emphasized that CATs should replace existing mandatory PROMs rather than supplement them. Some saw potential for more uniform PROM use across the healthcare chain: *“It would be helpful to see pre- and postoperative PROMIS CAT results to understand how patients return to us”* (PT#9). Also, PTs found it important to include PROMIS measures in professional clinical guidelines: *“If you want to implement something like this, it must be supported by the profession and included in guidelines. If it’s not widely backed and doesn’t replace existing* PROM*s, implementation will be difficult.” (*PT*#7).*Although PTs understood that maintaining PROMIS requires financial investment, most were unwilling to pay out of pocket. Practice owners were open to paying only if CATs provided clear time savings: *“If it clearly saves time for my therapists, then I would consider paying for the CATs.”* (PT#6).

### User-driven improvements

Some patients wanted more transparency and control over their responses. One patient said, *“A graph is fine, but seeing the individual questions would show more clearly what you can and cannot do”* (Patient#9). PTs agreed that viewing item-level responses would aid interviewing and goal setting: *“When it’s filled out online beforehand, you miss discussing the individual items together”* (PT#5). Patients also wished to review and correct their responses before final submission. Additional domains, such as lower extremity function, anxiety, depression, pain behavior, self-efficacy, social roles, fatigue, and sleep disturbance, were suggested as relevant for primary care physical therapy (Table 7).

### Integrated summary

Survey and interview findings converged in showing that implementation of PROMIS CATs was feasible but not yet structurally embedded within clinical practice. Although PTs generally felt confident interpreting and applying CAT scores, the interviews clarified that many still required further training on T-scores, cut-offs, MIC/MDC, and practical use. The interview findings further identified replacement of mandatory legacy PROMs, professional and organizational support, sustainable financing, and greater access to item-level information as important conditions for wider implementation.

## Discussion

In this study we examined the feasibility of Dutch-Flemish PROMIS CATs in primary care physical therapy for patients with MSK conditions of the spine and upper extremity, using Bowen’s feasibility domains of acceptability, demand, practicality, and implementation. Overall, PROMIS CATs were viewed as acceptable, and there was clear interest among PTs in using them more routinely, although actual uptake in daily practice was limited. Both PTs and patients considered the CATs efficient and practical to administer, and both groups provided valuable suggestions for optimizing future implementation.

Our findings are broadly consistent with recent systematic reviews on the use of PROMs in routine care (i.e. PROMs in general, rather than PROMIS CATs), which show that PROM-systems are generally acceptable and can be integrated into clinical workflows when supported by clear feedback and feasible administration processes [2,16]. These reviews highlight that clinicians require understandable, actionable feedback to inform clinical reasoning, while patients need clear explanations of their PROM results to perceive PROMs as meaningful rather than burdensome. Qualitative syntheses suggest that PROMs help clinicians identify patients’ needs and support more person-centered interactions, but also note barriers such as workflow disruption, questionnaire burden and variability in perceived relevance [2]. Patient-focused reviews similarly highlight that PROMs are useful when results are discussed during consultations, although long or repetitive questionnaires may reduce engagement [36]. The Cochrane review by Gibbons et al. (2021) further concludes that routine PROM-feedback can improve communication and satisfaction, though implementation success depends on usability and workflow alignment [17]. Evidence from PROM-portal studies in hospital care mirrors these themes. Teela et al. (2021) and van Muilekom et al. (2022) found that PROM-based conversations were valued but sensitive to questionnaire burden and workflow demands, reinforcing the need for shorter and more efficient measures such as PROMIS CATs [19,20]. Together, these findings support the feasibility of using PROMs in primary care physical therapy and align with several of the barriers and facilitators identified in our study.

Focusing more specifically on PROMIS CATs, several earlier studies have described their added value over legacy PROMs. In broader primary care populations, PROMIS CATs have been shown to support clinical decision-making, although these studies did not examine integration into routine workflows [37]. Across rheumatology, oncology and orthopedics, PROMIS CATs have demonstrated high completion rates, short administration times, and good acceptability, even in busy outpatient settings [38–41]. Large-scale implementation in orthopedic care has shown that CATs can be integrated into electronic medical records with minimal impact on clinic throughput [40]. Ambulatory sports medicine studies similarly report high compliance and low administrative burden [41]. In surgical MSK populations, PROMIS CATs consistently require fewer items than instruments such as the Knee Injury and Osteoarthritis Outcome Score (KOOS) or Western Ontario and McMaster Universities Osteoarthritis Index (WOMAC) while maintaining comparable precision and strong completion rates [42,43]. These results highlight the efficiency advantages of CAT administration, which is particularly relevant for primary care PTs working under time constraints. Work from inpatient rehabilitation further underscores that while technical feasibility is high, successful implementation depends on staff support, digital infrastructure, and workflow planning [44].

Taken together, existing evidence demonstrates strong feasibility of PROMIS CATs in specialist, surgical, and rehabilitation contexts. However, no studies to date have examined PROMIS CATs in primary care physical therapy. Our study therefore extends the literature by evaluating feasibility in primary care physical therapy practice and by integrating both quantitative and qualitative data on usability, workflow integration, and clinician and patient acceptability.

Complementing the current evidence, several specific findings from our study warrant further reflection. First, some PTs expressed reluctance to discuss graphical feedback, particularly red color coding and labels such as “severe”, due to concerns about potential nocebo effects. This contrasts with the Cochrane review by Gibbons et al. (2021), which found no evidence of adverse effects such as distress from PROM completion [17], and with findings from van Muilekom et al. (2021), who reported that line graphs with cut-off lines and color coding were preferred [25]. Whether PT concerns accurately reflect patient experiences remains uncertain. Second, PTs reported that administering and discussing CATs added time burden yet also described CATs as quick and easy. This discrepancy likely reflects the design of the present study, in which CATs were administered alongside mandatory legacy PROMs, increasing overall respondent burden. Third, some PTs and patients perceived several UE CAT items as repetitive or insufficient specific and experienced the UE CAT as relative long. The number of items administered during individual CAT assessments was not available in the dataset used for this feasibility study; therefore these perceptions could not be compared with objective CAT administration data. The perception that the UE CAT was relative long, may reduce engagement. In addition, reports that some items were repetitive or insufficiently specific may indicate potential limitations in the content validity of the Dutch-Flemish UE item bank for this target population, warranting further investigation [45]. Finally, both PTs and patients expressed interest in viewing individual item responses, which aligns with findings by Teela et al. (2021) and van Muilekom et al. (2022) showing that clinicians and patients valued item-level feedback [19,20]. An explanation for the desire to view individual item responses may lie in PTs’ long-standing use of patient-specific instruments, such as the PSFS, in which patients identify one or more priority activities that then guide goal setting and treatment evaluation. Item-level feedback may intuitively feel closer to this familiar way of working. Viewing individual item responses may support clinical conversations; however, item-level changes over time are not presented, as such comparisons are not considered reliable in CAT-based assessments due to variation in administered items.

This study has several strengths. PROMIS CATs were fully integrated into the EHR-system and incorporated into routine workflows, allowing evaluation under real-world conditions rather than controlled research settings. The mixed-methods design, combining a therapist survey with interviews from both PTs and patients, provided a multidimensional understanding of acceptability, perceived value, and practical challenges. Finally, the involvement of 14 practices, 25 PTs, and nine patients provide a broad representation across Dutch primary care physical therapy, enhancing contextual generalizability.

Limitations should also be acknowledged. First, although purposive sampling for interviews was planned, limited willingness among potential participants resulted in reliance on convenience sampling, potentially underrepresenting individuals with lower digital literacy, limited health literacy, or language barriers. Second, patients were required to complete both legacy PROMs and PROMIS CATs, increasing total PROM respondent burden and possibly influencing PT perceptions of workload. Future studies should examine workflows in which CATs fully replace legacy instruments and include comparison groups using traditional PROMs. Third, despite efforts to clarify distinctions between legacy PROMs and PROMIS CATs (e.g., simulating CAT administration before interviews), participants may not always have differentiated clearly between them in their responses to the survey or discussing the barriers or facilitators for CATs in the interviews.

The results showed an interest among PTs in a broader range of PROMIS-domains relevant to primary care physical therapy, such as lower extremity function, anxiety, depression, pain behavior, self-efficacy, social roles, fatigue, and sleep disturbance, which warrants further research. Building on recent pediatric physical therapy work, aimed at defining a core set of PROs and PROMs [46], the development of a corresponding core set for adults appears warranted. A core set of generic PROMs has already been recommended for adults in medical specialist care [47], but its applicability or modification to adult care physical therapy remains to be determined.

Broader implementation of PROMIS CATs will also require addressing technical and financial considerations. Real-time CAT administration depends on API integration with EHR-systems, and the diverse EHR landscape in Dutch primary care means that vendors will need to invest in this infrastructure. Fees for CATs remain an unresolved issue in a sector with constrained reimbursement. PROMIS short forms may offer a cost-efficient alternative, not requiring API integration, though their feasibility and added value in primary care physical therapy remain to be evaluated. Uptake may also be supported if national quality registries such as the Association for Quality in Physical Therapy (SKF) and the Royal Dutch Society for Physical Therapy (KNGF) recommend generic PROMIS measures as an alternative for disease specific legacy instruments and incorporate them into clinical guidelines. Likely, combining PROMIS domain scores with a structured patient-specific goal-setting approach (such as using the PSFS), may be valuable in routine practice. Such an approach could preserve the efficiency and comprehensiveness of PROMIS CATs and allow their scores to be used for quality evaluation at an aggregated level, while supporting the individualized goal-setting processes that PTs consider essential in daily practice. Finally, training for PTs on interpreting PROMIS (CAT) scores, including MDC, MIC and interpretability, and integrating PROM data into clinical decision-making processes will be essential for sustainable implementation.

## Conclusions

In summary, this study demonstrates that implementing PROMIS CATs in primary care physical therapy is feasible and well-received by both PTs and patients, under conditions. When integrated into routine workflows, PROMIS CATs support structured and patient-centered conversations about symptoms and functioning, while maintaining acceptable respondent burden. Sustainable adoption will require improved technical integration, clinician training, appropriate financial reimbursement and support from health insurers, and incorporation of PROMIS into clinical guidelines. Future research should address the development of a core set of generic PROs and PROMs for adult physical therapy care.

## Supporting information

S1 Table Key areas of focus according to Bowen et al. (2009) with interpretation in the context of this study. (DOCX)

S2 Table COREQ 32- item Checklist for Qualitative studies. (DOCX)

S3 Table Survey on the use of PROMIS CAT by physical therapists in the Scoring Smarter study. (DOCX)

S4 Table Final interview guide for patients and physical therapists. (DOCX)

## References

[1] Black N. Patient reported outcome measures could help transform healthcare. BMJ. 2013;346:f167. doi: 10.1136/bmj.f167 23358487

[2] Boyce MB, Browne JP, Greenhalgh J. The experiences of professionals with using information from patient-reported outcome measures to improve the quality of healthcare: a systematic review of qualitative research. BMJ Qual Saf. 2014;23(6):508–18. doi: 10.1136/bmjqs-2013-002524 24505110

[3] Cella D, Gershon R, Lai J-S, Choi S. The future of outcomes measurement: item banking, tailored short-forms, and computerized adaptive assessment. Qual Life Res. 2007;16 Suppl 1:133–41. doi: 10.1007/s11136-007-9204-6 17401637

[4] Cella D, Riley W, Stone A, Rothrock N, Reeve B, Yount S, et al. The Patient-Reported Outcomes Measurement Information System (PROMIS) developed and tested its first wave of adult self-reported health outcome item banks: 2005-2008. J Clin Epidemiol. 2010;63(11):1179–94. doi: 10.1016/j.jclinepi.2010.04.011 20685078 PMC2965562

[5] Fries JF, Cella D, Rose M, Krishnan E, Bruce B. Progress in assessing physical function in arthritis: PROMIS short forms and computerized adaptive testing. J Rheumatol. 2009;36(9):2061–6. doi: 10.3899/jrheum.090358 19738214

[6] Revicki DA, Chen W-H, Harnam N, Cook KF, Amtmann D, Callahan LF, et al. Development and psychometric analysis of the PROMIS pain behavior item bank. Pain. 2009;146(1–2):158–69. doi: 10.1016/j.pain.2009.07.029 19683873 PMC2775487

[7] Rose M, Bjorner JB, Gandek B, Bruce B, Fries JF, Ware JE Jr. The PROMIS Physical Function item bank was calibrated to a standardized metric and shown to improve measurement efficiency. J Clin Epidemiol. 2014;67(5):516–26. doi: 10.1016/j.jclinepi.2013.10.024 24698295 PMC4465404

[8] Terwee CB, Roorda LD, de Vet HCW, Dekker J, Westhovens R, van Leeuwen J, et al. Dutch-Flemish translation of 17 item banks from the patient-reported outcomes measurement information system (PROMIS). Qual Life Res. 2014;23(6):1733–41. doi: 10.1007/s11136-013-0611-6 24402179

[9] Crins MHP, Roorda LD, Smits N, de Vet HCW, Westhovens R, Cella D, et al. Calibration and validation of the dutch-flemish PROMIS Pain interference item bank in patients with chronic pain. PLoS One. 2015;10(7):e0134094. doi: 10.1371/journal.pone.0134094 26214178 PMC4516300

[10] Crins MHP, van der Wees PJ, Klausch T, van Dulmen SA, Roorda LD, Terwee CB. Psychometric properties of the PROMIS Physical Function item bank in patients receiving physical therapy. PLoS One. 2018;13(2):e0192187. doi: 10.1371/journal.pone.0192187 29432433 PMC5809015

[11] Haan E-JA, Terwee CB, Van Wier MF, Willigenburg NW, Van Deurzen DFP, Pisters MF, et al. Translation, cross-cultural and construct validity of the Dutch-Flemish PROMIS® upper extremity item bank v2.0. Qual Life Res. 2020;29(4):1123–35. doi: 10.1007/s11136-019-02388-2 31894506

[12] van Bruggen SGJ, Lameijer CM, Terwee CB. Structural validity and construct validity of the Dutch-Flemish PROMIS® physical function-upper extremity version 2.0 item bank in Dutch patients with upper extremity injuries. Disabil Rehabil. 2021;43(8):1176–84. doi: 10.1080/09638288.2019.1651908 31411908

[13] Lameijer CM, van Bruggen SGJ, Haan EJA, Van Deurzen DFP, Van der Elst K, Stouten V, et al. Graded response model fit, measurement invariance and (comparative) precision of the Dutch-Flemish PROMIS® Upper Extremity V2.0 item bank in patients with upper extremity disorders. BMC Musculoskelet Disord. 2020;21(1):170. doi: 10.1186/s12891-020-3178-8 32178644 PMC7077019

[14] Arensman RM, Haan EJA, Terwee CB, van Rosmalen J, Wittink H, Kiers H. Test-retest reliability and minimal detectable change of Dutch-Flemish Patient Reported Outcomes Measurement Information System (PROMIS®) computerized adaptive tests for musculoskeletal disorders. PLoS One. 2025;20(10):e0333670. doi: 10.1371/journal.pone.0333670 41071775 PMC12513665

[15] Arensman RM, Haan EJA, Terluin B, Terwee CB, Wittink H, Kiers H. Responsiveness and minimal important change of Dutch-Flemish Patient reported outcomes measurement information system (PROMIS®) computerized adaptive tests for musculoskeletal disorders. Advances in Patient-Reported Outcomes. 2025;1(2):100198. doi: 10.1016/j.apro.2025.100198

[16] Campbell R, Ju A, King MT, Rutherford C. Perceived benefits and limitations of using patient-reported outcome measures in clinical practice with individual patients: A systematic review of qualitative studies. Qual Life Res. 2022;31(6):1597–620. doi: 10.1007/s11136-021-03003-z 34580822

[17] Gibbons C, Porter I, Gonçalves-Bradley DC, Stoilov S, Ricci-Cabello I, Tsangaris E, et al. Routine provision of feedback from patient-reported outcome measurements to healthcare providers and patients in clinical practice. Cochrane Database Syst Rev. 2021;10(10):CD011589. doi: 10.1002/14651858.CD011589.pub2 34637526 PMC8509115

[18] Greenhalgh J, Gooding K, Gibbons E, Dalkin S, Wright J, Valderas J, et al. How do patient reported outcome measures (PROMs) support clinician-patient communication and patient care? A realist synthesis. J Patient Rep Outcomes. 2018;2:42. doi: 10.1186/s41687-018-0061-6 30294712 PMC6153194

[19] Teela L, van Muilekom MM, Kooij LH, Gathier AW, van Goudoever JB, Grootenhuis MA, et al. Clinicians’ perspective on the implemented KLIK PROM portal in clinical practice. Qual Life Res. 2021;30(11):3267–77. doi: 10.1007/s11136-020-02522-5 32468402 PMC8528749

[20] van Muilekom MM, Teela L, van Oers HA, van Goudoever JB, Grootenhuis MA, Haverman L. Patients’ and parents’ perspective on the implementation of patient reported outcome measures in pediatric clinical practice using the KLIK PROM portal. Qual Life Res. 2022;31(1):241–54. doi: 10.1007/s11136-021-02950-x 34324137 PMC8800898

[21] Bowen DJ, Kreuter M, Spring B, Cofta-Woerpel L, Linnan L, Weiner D, et al. How we design feasibility studies. Am J Prev Med. 2009;36(5):452–7. doi: 10.1016/j.amepre.2009.02.002 19362699 PMC2859314

[22] Bishop FL. Using mixed methods research designs in health psychology: an illustrated discussion from a pragmatist perspective. Br J Health Psychol. 2015;20(1):5–20. doi: 10.1111/bjhp.12122 25405549

[23] Tong A, Sainsbury P, Craig J. Consolidated criteria for reporting qualitative research (COREQ): A 32-item checklist for interviews and focus groups. Int J Qual Health Care. 2007;19(6):349–57. doi: 10.1093/intqhc/mzm042 17872937

[24] Palinkas LA, Horwitz SM, Green CA, Wisdom JP, Duan N, Hoagwood K. Purposeful sampling for qualitative data collection and analysis in mixed method implementation research. Adm Policy Ment Health. 2015;42(5):533–44. doi: 10.1007/s10488-013-0528-y 24193818 PMC4012002

[25] van Muilekom MM, Luijten MAJ, van Oers HA, Terwee CB, van Litsenburg RRL, Roorda LD, et al. From statistics to clinics: The visual feedback of PROMIS® CATs. J Patient Rep Outcomes. 2021;5(1):55. doi: 10.1186/s41687-021-00324-y 34245390 PMC8272760

[26] Chiarotto A, Maxwell LJ, Ostelo RW, Boers M, Tugwell P, Terwee CB. Measurement properties of visual analogue scale, numeric rating scale, and pain severity subscale of the brief pain inventory in patients with low back pain: A systematic review. J Pain. 2019;20(3):245–63. doi: 10.1016/j.jpain.2018.07.009 30099210

[27] Thoomes E, Cleland JA, Falla D, Bier J, de Graaf M. Reliability, measurement error, responsiveness, and minimal important change of the patient-specific functional scale 2.0 for patients with nonspecific neck pain. Phys Ther. 2024;104(1). doi: 10.1093/ptj/pzad113 37606246 PMC10776311

[28] Speksnijder CM, Koppenaal T, Knottnerus JA, Spigt M, Staal JB, Terwee CB. Measurement properties of the quebec back pain disability scale in patients with nonspecific low back pain: Systematic review. Phys Ther. 2016;96(11):1816–31. doi: 10.2522/ptj.20140478 27231271

[29] MacDermid JC, Walton DM, Avery S, Blanchard A, Etruw E, McAlpine C, et al. Measurement properties of the neck disability index: A systematic review. J Orthop Sports Phys Ther. 2009;39(5):400–17. doi: 10.2519/jospt.2009.2930 19521015

[30] Kennedy CA, Beaton DE, Smith P, Van Eerd D, Tang K, Inrig T, et al. Measurement properties of the QuickDASH (disabilities of the arm, shoulder and hand) outcome measure and cross-cultural adaptations of the QuickDASH: A systematic review. Qual Life Res. 2013;22(9):2509–47. doi: 10.1007/s11136-013-0362-4 23479209

[31] Meerhoff GA, van Dulmen SA, Maas MJM, Heijblom K, Nijhuis-van der Sanden MWG, Van der Wees PJ. Development and evaluation of an implementation strategy for collecting data in a national registry and the use of patient-reported outcome measures in physical therapist practices: Quality improvement study. Phys Ther. 2017;97(8):837–51. doi: 10.1093/ptj/pzx051 28789466

[32] Braun V, Clarke V. Using thematic analysis in psychology. Qualitative Res Psychol. 2006;3(2):77–101. doi: 10.1191/1478088706qp063oa

[33] Fereday J, Muir-Cochrane E. Demonstrating rigor using thematic analysis: A hybrid approach of inductive and deductive coding and theme development. Int J Qualitative Methods. 2006;5(1):80–92. doi: 10.1177/160940690600500107

[34] Saunders B, Sim J, Kingstone T, Baker S, Waterfield J, Bartlam B, et al. Saturation in qualitative research: exploring its conceptualization and operationalization. Qual Quant. 2018;52(4):1893–907. doi: 10.1007/s11135-017-0574-8 29937585 PMC5993836

[35] Hennink M, Kaiser BN. Sample sizes for saturation in qualitative research: A systematic review of empirical tests. Soc Sci Med. 2022;292:114523. doi: 10.1016/j.socscimed.2021.114523 34785096

[36] Carfora L, Foley CM, Hagi-Diakou P, Lesty PJ, Sandstrom ML, Ramsey I, et al. Patients’ experiences and perspectives of patient-reported outcome measures in clinical care: A systematic review and qualitative meta-synthesis. PLoS One. 2022;17(4):e0267030. doi: 10.1371/journal.pone.0267030 35446885 PMC9022863

[37] Jacobson RP, Kang D, Houck J. Can patient-reported outcomes measurement information system(R) (PROMIS) measures accurately enhance understanding of acceptable symptoms and functioning in primary care?. J Patient Rep Outcomes. 2020;4(1):39. doi: 10.1186/s41687-020-00206-9 32436001 PMC7239962

[38] Kasturi S, Burket JC, Berman JR, Kirou KA, Levine AB, Sammaritano LR, et al. Feasibility of patient-reported outcomes measurement information system (PROMIS®) computerized adaptive tests in systemic lupus erythematosus outpatients. Lupus. 2018;27(10):1591–9. doi: 10.1177/0961203318778372 29793381

[39] Leung YW, Brown C, Cosio AP, Dobriyal A, Malik N, Pat V, et al. Feasibility and diagnostic accuracy of the patient-reported outcomes measurement information system (PROMIS) item banks for routine surveillance of sleep and fatigue problems in ambulatory cancer care. Cancer. 2016;122(18):2906–17. doi: 10.1002/cncr.30134 27351521

[40] Papuga MO, Dasilva C, McIntyre A, Mitten D, Kates S, Baumhauer JF. Large-scale clinical implementation of PROMIS computer adaptive testing with direct incorporation into the electronic medical record. Health Syst (Basingstoke). 2017;7(1):1–12. doi: 10.1057/s41306-016-0016-1 31214335 PMC6452834

[41] Lizzio VA, Blanchett J, Borowsky P, Meldau JE, Verma NN, Muh S, et al. Feasibility of PROMIS CAT administration in the ambulatory sports medicine clinic with respect to cost and patient compliance: A single-surgeon experience. Orthop J Sports Med. 2019;7(1):2325967118821875. doi: 10.1177/2325967118821875 30733973 PMC6344951

[42] Braaksma C, Wolterbeek N, Veen MR, Poolman RW, Pronk Y, Klaassen AD, et al. Assessing the measurement properties of PROMIS Computer Adaptive Tests, short forms and legacy patient reported outcome measures in patients undergoing total hip arthroplasty. J Patient Rep Outcomes. 2024;8(1):121. doi: 10.1186/s41687-024-00799-5 39432233 PMC11493881

[43] Dhollander O, Roorda LD, Diarra S, Ghijselings I, Demurie A, Terwee CB, et al. Psychometric properties and feasibility of PROMIS computerized adaptive tests compared with disease-specific measures in knee arthroplasty. J Bone Joint Surg Am. 2025;107(21):2371–88. doi: 10.2106/JBJS.24.01348 40956870 PMC12573681

[44] Rafiq RB, Yount S, Jerousek S, Roth EJ, Cella D, Albert MV, et al. Feasibility of PROMIS using computerized adaptive testing during inpatient rehabilitation. J Patient Rep Outcomes. 2023;7(1):44. doi: 10.1186/s41687-023-00567-x 37162607 PMC10172423

[45] Abma IL, Butje BJD, Ten Klooster PM, van der Wees PJ. Measurement properties of the Dutch-Flemish patient-reported outcomes measurement information system (PROMIS) physical function item bank and instruments: A systematic review. Health Qual Life Outcomes. 2021;19(1):62. doi: 10.1186/s12955-020-01647-y 33627157 PMC7905571

[46] Korteling DL, Limmen S, Ketelaar M, Daams JG, Luijten MAJ, van Oers HA, et al. Patient reported outcomes in pediatric physical therapy: A scoping review and evidence map. J Patient Rep Outcomes. 2025;9(1):125. doi: 10.1186/s41687-025-00947-5 41134430 PMC12552199

[47] Oude Voshaar M, Terwee CB, Haverman L, van der Kolk B, Harkes M, van Woerden CS, et al. Development of a standard set of PROs and generic PROMs for Dutch medical specialist care : Recommendations from the outcome-based healthcare program working group generic PROMs. Qual Life Res. 2023;32(6):1595–605. doi: 10.1007/s11136-022-03328-3 36757571 PMC10172289

