# Supplementary material for: Feasibility of patient-reported outcomes measurement information system (PROMIS) computerized adaptive tests in patients receiving physical therapy: A mixed-methods study
Source: PLoS One. 2026 Nov 13;21(11):e0362652. doi: 10.1371/journal.pone.0362652 (PMC13766268; doi:10.1371/journal.pone.0362652)
Supplement: Supplementary file 1 [file pone.0362652.s001.docx]

**Key areas of focus according to Bowen et al. (2009) with interpretation in the context of this study.**

| Area of focus | Interpretation in the context of this study |
| --- | --- |
| Acceptability | Acceptability addresses the question whether the application of the PROMIS CAT will be suitable, satisfying or attractive to patients and therapists. Acceptability can be expressed by participants as satisfaction, intent to continue use, perceived appropriateness and fit within and effects on organizational factors. |
| Demand | Demand could comprise the actual use of and the expressed interest in the PROMIS CAT, the intention to use the PROMIS CAT and the perceived demand for the new concept of constructing and presenting PROMs. |
| Practicality | Practicality is the ability of participants to provide and complete the PROMIS CAT, the positive or negative effects on participants and the ability of therapists to interpret and apply the results within existing digital resources and with existing other means, resources and circumstances. |
| Implementation | To what extent could PROMIS CATs successfully be implemented in the context of primary care physical therapy practice. What is the actual degree of execution, the success or failure of execution. What are the amount and type of resources needed to implement the PROMIS CAT. Which are factors that affect implementation ease or difficulty. What is the efficiency, speed, or quality of implementation of PROMIS CAT. |
