# Supplementary material for: Feasibility of patient-reported outcomes measurement information system (PROMIS) computerized adaptive tests in patients receiving physical therapy: A mixed-methods study
Source: PLoS One. 2026 Nov 13;21(11):e0362652. doi: 10.1371/journal.pone.0362652 (PMC13766268; doi:10.1371/journal.pone.0362652)
Supplement: Supplementary file 2 [file pone.0362652.s002.docx]

**COREQ 32- item Checklist for Qualitative studies.**

| **No. Item** | **Guide Question/description** |  |
| --- | --- | --- |
| **Domain 1 Research team and reflexivity** | | |
| **Personal Characteristics** | | |
| **1.**Interviewer/facilitator | Which author/s conducted the interview or focus group? | First author (EH) and PTs AJ, EM, MK, HA, and JO. |
| **2.** Credentials | What were the researcher's credentials? | MSc (EH, AD and AJ) and MSc third year students (EM, HA, MK and JO) and PTs. |
| **3.** Occupation | What was their occupation at the time of the study? | Phd student and PT MT in PT practice (EH). Phd student (AD). MSc pediatric PT (AJ), Mental health PT (EM), sports PT (HA) and MSc second year student Master Physical Therapy and Innovation in Movement Care (EM and HA) and MSc third year students in MT and working in PT practice (MK and JO). |
| 4. Gender | Was the researcher male or female? | Males (EH, MK, JO) and females (AD, AJ, EM, HA). |
| **5.** Experience and training | What experience or training did the researcher have? | Qualitative research courses with practical exercise in interviewing and data analysis.  Specific for this study; two pilot interviews with observation of experienced interviewers. |
| **Relationship with participants** | | |
| **6.** Relationship established | Was a relationship established prior to study commencement? | The participants only had contact by email before the interviews. |
| **7.** Participant knowledge of the interviewer | What did the participants know about the researcher? e.g. personal goals, reasons for doing the research? | Three therapist participants (T01, T03 and T05) worked in the same private practice as the first author (EH). All other therapist participants and patient participants did not have personal relationships with the researchers. An introduction email was sent with information about the purpose of the research. |
| **8.** Interviewer characteristics | What characteristics were reported about the interviewer/facilitator? e.g. Bias, assumptions, reasons and interests in the research topic. | All interviews were conducted by a researcher and a PT*.* The lead interviewer (EH) was a male researcher and PT MT with 24 years of work experience in private practice and chronic pain rehabilitation setting and had a clinical and research interest in PROMs and PROMIS. AJ was a pediatric PT with four years working experience in private practice and experience as a research assistant in qualitative research. EM, HA, MK and JO were PTs and MSc students, working on their thesis with this study as their topic, and had 15, 8, 4 and 8 years of work experience in private practice, respectively. |
| **Domain: study design** | | |
| *Theoretical framework* | | |
| 9. Methodological orientation and Theory | What methodological orientation was started to underpin the study? E.g. grounded theory, discourse analysis, ethnography, phenomenology, content analysis | Qualitative content analysis. |
| *Participant selection* | | |
| 10. Sampling | How were participants selected? E.g. purposive, convenience, consecutive, snowball | Convenience sampling. |
| 11. Method of approach | How were participants approached? E.g. face-to-face, telephone, mail, email | Patients were approached face to face by their PT; PTs were approached by email. |
| 12. Sample size | How many participants were in the study? | 19 participants in the interviews and 25 participants in the survey. |
| 13. Non-participation | How many people refused to participate or dropped out? Reasons? | 51 participants were invited for the survey, of which 25 completed the survey. For the interviews no participants refused or dropped out. |
| *Setting* | | |
| 14. Setting of data collection | Where was the data collected? E.g. home, clinic, workplace | The data were collected through an online secured data collection tool (Crowdtech^®^) for the survey, and through MS Teams for the interviews. |
| 15. Presence of non-participants | Was anyone else present besides the participants and researchers? | No. Thirteen interviews were held with two interviewers, in six interviews (P01, P02, P03, T02, T05 and T06) there was a third PT present. |
| 16. Description of sample | What are the important characteristics of the sample? E.g. demographic data, date | Eleven males and fourteen females, mean age 39 (range 27 - 63) years, participated in the survey.  Seven males and twelve females, mean age 40 (range 22–70) years, participated in the interview study. See Table 1a and 1b in the results section. |
| *Data collection* | | |
| 17. Interview guide | Were questions, prompts, guides provided by the authors? Was it pilot tested? | The interview guide was developed in advance by the research team. Questions were developed based on a previous study which examined implementation of PROMs (Meerhoff et al. 2017), another study that examined clinicians’ perspectives on a patient PROM portal (Teela et al. 2021), the clinical experience of the research team, and the areas of focus for feasibility studies (Bowen et al. 2009). Three pilot interviews were audiotaped, transcribed, and reviewed by the first author to refine the interview guide further. |
| 18. Repeat interviews | Were repeat interviews carried out? If yes, how many? | No |
| 19. Audio/visual recording | Did the research use audio or visual recording to collect the data? | Audio recording. |
| 20. Field notes | Were field notes made during and/or after the interview or focus group? | Field notes were made by the second interviewer. Focused on non-verbal interaction. |
| 21. Duration | What was the duration of the interviews or focus group? | The interviews lasted between 32 and 62 minutes (mean = 47 min, SD = 8 min). |
| 22. Data saturation | Was data saturation discussed? | No. |
| 23. Transcripts returned | Where transcripts returned to participants for comment and/or correction? | The participants were invited to comment on the transcripts. None of the participants was interested to provide comment and/or correction. |
| **Domain 3 Analysis and findings** | | |
| *Data analysis* | | |
| 24. Number of data coders | How many data coders coded the data? | There were three data coders involved. Every interview description was coded by two data coders. |
| 25. Description of the coding tree | Did authors provide a description of the coding tree? | After the first interview with a patient and the first interview with a therapist, two researchers (EH and AJ) open-coded the text line by line, following a group meeting to discuss and defined the open codes. Subsequently, all interviews were independently open-coded by EH and AJ or AD following a consensus meeting.  Every second interview was compared with the previous analysis to identify similarities and differences were discussed between the coders. |
| 26. Derivation of themes | Were themes identified in advance or derived from the data? | The themes derived from the data and themes were then organized deductively within the four domains of Bowen’s feasibility framework. |
| 27. Software | What software, if applicable, was used to manage the data? | Amberscript for transcription of the interviews and Atlas.ti for support in the data-analysis. |
| 28. Participant checking | Did participants provide feedback on the findings? | They were invited to but none of the participant requested changes. |
| *Reporting* | | |
| 29. Quotations presented | Were participant quotations presented to illustrate the themes/findings? Was each quotations identified? E.g. participant number | Yes See quotations in the text in the result section. |
| 30. Data and findings consistent | Was there consistency between the data presented and the findings? | Yes See Table 4 and text in the result section. |
| 31. Clarity of major themes | Were major themes clearly presented in the findings? | Yes See Table 4 and text in the result section. |
| 32. Clarity of minor themes | Is there a description of diverse cases or discussion of minor themes? | Yes, briefly. See text in the results and discussion section. |
