# Supplementary material for: Feasibility of patient-reported outcomes measurement information system (PROMIS) computerized adaptive tests in patients receiving physical therapy: A mixed-methods study
Source: PLoS One. 2026 Nov 13;21(11):e0362652. doi: 10.1371/journal.pone.0362652 (PMC13766268; doi:10.1371/journal.pone.0362652)
Supplement: Supplementary file 3 [file pone.0362652.s003.docx]

**Survey on the use of PROMIS CAT by physical therapists in the Scoring Smarter study.**

Dear colleague,

You are participating in the Scoring Smarter study. In this study, we are looking at applying the Patient Reported Outcomes Measurement Information System (PROMIS) in primary care physical therapy. PROMIS is a measurement system where patient-reported outcomes (PROs) can be queried smartly and efficiently utilizing a Computer Adaptive Test (CAT). This involves the computer choosing a question relevant to the patient each time based on the patient’s answer to the previous question. During the Scoring Smarter study, you administered PROMIS CATs to patients in their treatment trajectory. Through the following questions, we would like to hear your opinion on, and thus gain insight into, your experiences with the use of PROMIS CATs in treating patients with musculoskeletal complaints.

Completing the questionnaire takes about 10-15 minutes.

| 1. | I have read the information letter. I declare that I was informed in a way that was clear to me about the nature, method, purpose and burden of the study. I was able to ask my questions and had enough time to decide whether to participate.  I know that the data and results of the study, for the purposes stated in the information letter, will only be disclosed to third parties pseudonymously and confidentially.  I consent to the pseudonymized collection, retention and use of my data for the purpose of answering the research question in the study.  I want to participate in this study, and I know that my data will be protected pseudonymously.  I know that participating is voluntary and that I can stop at any time during the study. I do not have to give a reason for this. I know that the already collected data will be used. | | | | | | | |
| --- | --- | --- | --- | --- | --- | --- | --- | --- |
|  | **Part 1. Questions about using PROMIS CAT** | | | | | | | |
|  |  | Completely agree | Agree | Don’t agree / don’t disagree | Disagree | Completely  disagree |  | **Measuring the area** |
| 2. | I am interested in using PROMIS CATs. | O | O | O | O | O |  | Demand |
| 3. | I know where to find PROMIS CATs in my Electronic Health Record (EHR). | O | O | O | O | O |  | Practicality |
| 4. | I am able to administer PROMIS CATs to my patients. | O | O | O | O | O |  | Practicality |
| 5. | I am able to correctly interpret PROMIS CAT scores. | O | O | O | O | O |  | Practicality |
| 6. | Using PROMIS CATs does not affect my professional authority to make my own decisions. | O | O | O | O | O |  | Acceptability |
| 7. | I find that PROMIS CAT scores helps me to formulate a physical therapy diagnosis. | O | O | O | O | O |  | Acceptability |
| 8. | I find that a PROMIS CAT score contributes to the evaluation of physical therapy treatment. | O | O | O | O | O |  | Acceptability |
| 9. | I know how to apply PROMIS CATs within my clinical reasoning process. | O | O | O | O | O |  | Practicality |
| 10. | I find that the use of PROMIS CATs contributes to the quality of physical therapy healthcare. | O | O | O | O | O |  | Acceptability |
| 11. | I find it important to objectively capture patient data through PROMIS CATs. | O | O | O | O | O |  | Demand |
| 12. | I believe that the use of PROMIS CATs should be a part of physical therapy clinical reasoning. | O | O | O | O | O |  | Demand |
| 13. | I find that working with PROMIS CATs takes little time. | O | O | O | O | O |  | Practicality |
| 14. | Patients find that using PROMIS CATs takes little time. | O | O | O | O | O |  | Practicality |
| 15. | When using PROMIS CATs I am able to include my patients’ wishes. | O | O | O | O | O |  | Acceptability |
| 16. | I have a positive attitude towards the use of PROMIS CATs. | O | O | O | O | O |  | Acceptability |
| 17. | I would like to use PROMIS CATs more often in my practice. | O | O | O | O | O |  | Demand |
| 18. | I use PROMIS CATs in daily practice. | O | O | O | O | O |  | Demand |
| 19. | I have experienced the added value of using PROMIS CATs. | O | O | O | O | O |  | Acceptability |
| 20. | Patients are cooperative in using PROMIS CATs. | O | O | O | O | O |  | Practicality |
| 21. | Using PROMIS CATs is a standard part of my clinical reasoning. | O | O | O | O | O |  | Acceptability |
| 22. | I have sufficient routine in the use of PROMIS CATs. | O | O | O | O | O |  | Acceptability |
| **Part 1a. Specific questions about PROMIS CAT in relation to the consultation** | | | | | | | | |
|  | **Use of PROMIS CAT during the consultation** |  | | | | |  |  |
| 23. | I discuss the PROMIs CAT scores with patients: | 0 Always  0 Mostly  0 Sometimes  0 Almost never  0 Never | | | | |  | Practicality |
| 24. | If answer to 23 =mostly/sometimes/almost never/never: The reason that I do not always discuss the PROMIS CAT scores with patients is: | Free-text field | | | | |  | Practicality |
| 25. | I discuss the PROMIS CAT scores at the .. of the treatment trajectory (multiple response options possible). | 0 Beginning  0 Interim  0 End | | | | |  | Practicality |
| 26  27. | On average, I use .. minutes of treatment appointment time to discuss PROMIS CAT scores.  To what extent are you satisfied with this? | .. (0-30)  0 I am satisfied with this  0 I would like to spend more time discussing the PROMIS CAT scores  0 I would like to spend less time discussing the PROMIS CAT scores | | | | |  | Practicality |
| 28. | All patients are invited to complete the PROMIS CATs online. | Completely agree | Agree | Don’t agree / don’t disagree | Disagree | Completely  disagree |  | Practicality |
| 29. | Is answer to 28 is: completely disagree/disagree/don’t agree/disagree, then: The reason not everyone is invited is: | Free-text field | | | | |  | Practicality |
| 30. | I estimate that ..% of patients who complete the PROMIS CATs do so online. | 0% | 0-25% | 25-50% | 50-75% | 75-100% |  | Practicality |
| 31. | I find that PROMIS CAT can easily be administered during the consultation. | Completely agree | Agree | Don’t agree / don’t disagree | Disagree | Completely  disagree |  | Practicality |
| 32. | If answer to 31 is: completely disagree/disagree/don’t agree/disagree, then:  The reason a PROMIS CAT cannot easily be administered during the consultation is: | Free-text field | | | | |  | Practicality |
|  | **Influence of PROMIS CAT scores on the consultation** | | | | | | | |
| 33. | When I use PROMIS CATs, I identify patient problems faster. | Completely agree | Agree | Don’t agree / don’t disagree | Disagree | Completely  disagree |  | Practicality |
| 34. | Explanation: | Free-text field | | | | |  | Practicality |
| 35. | Benefits of PROMIS CATs are: | Free-text field | | | | |  | Acceptability |
| 36. | Disadvantages of PROMIS CATs are: | Free-text field | | | | |  | Acceptability |
|  |  | Completely agree | Agree | Don’t agree / don’t disagree | Disagree | Completely  disagree |  |  |
| 37. | I think patients are satisfied with the use of PROMIS CATs. | O | O | O | O | O |  | Acceptability |
| 38. | Benefits for patients to use PROMIS CATs are: | Free-text field | | | | |  | Acceptability |
| 39 | The most frequently heard comments from patients about PROMIS CATs are: | Free-text field | | | | |  | Acceptability |
| 40. | PROMIS CATs are easy to use. | Completely agree | Agree | Don’t agree / don’t disagree | Disagree | Completely  disagree |  | Practicality |
| 41. | The graphs of the PROMIS CAT scores have an attractive lay-out. | Completely agree | Agree | Don’t agree / don’t disagree | Disagree | Completely  disagree |  | Acceptability |
| 42 | I am satisfied with the PROMIS CAT Physical Functioning. | O | O | O | O | O | NA/ Did not use | Acceptability |
| 43 | I am satisfied with the PROMIS CAT Upper Extremity. | O | O | O | O | O | NA/ Did not use | Acceptability |
| 44 | I am satisfied with the PROMIS CAT Pain Interference. | O | O | O | O | O | NA/ Did not use | Acceptability |
| 45. | I am satisfied with the graphical feedback (display of scores over time and comparison with the T-score of 50 as an average for the general population) | O | O | O | O | O |  | Acceptability |
| 46. | I am satisfied with the cut-off lines and labels in the graph (mild, moderate, severe). | O | O | O | O | O |  | Acceptability |
| 47. | I would also like to see back the scores on the separate questions (items) of the CAT | O | O | O | O | O |  | Acceptability |
| 48. | I would also like to be able to use the following domains (for adults) as PROMIS CAT: (multiple answers possible) | 0 Anxiety  0 Depression  0 Fatigue  0 Pain Behavior  0 Physical Function – Mobility (lower extremity)  0 Sleep Disturbance  0 Sleep-Related Impairment  0 Ability to Participate in Social Roles and Activities  0 Satisfaction with Social Roles and Activities  0 Self-Efficacy for Managing Chronic Conditions – Manage Meds/Treatment  0 Self-Efficacy for Managing Chronic Conditions – Manage Symptoms | | | | |  |  |
|  | **Support and training needs** | Completely agree | Agree | Don’t agree / don’t disagree | Disagree | Completely  disagree |  |  |
| 49. | I know where to go with my questions about PROMIS and PROMIS CAT | O | O | O | O | O |  | Practicality |
| 50. | I need training on PROMIS and PROMIS CAT | O | O | O | O | O |  | Practicality |
| 51. | I would like to see the following topics covered in training: | Free-text field | | | | |  | Practicality |
| **Part 2. Questions about policies within your practice** | | | | | | | | |
|  |  | Completely agree | Agree | Don’t agree / don’t disagree | Disagree | Completely  disagree |  |  |
| 52. | Within our practice, we have agreed on how to use PROMIS CATs. | O | O | O | O | O |  | Acceptability |
| 53. | Management supports staff in the use of PROMIS CATs. | O | O | O | O | O |  | Implementation |
| 54. | Management itself uses PROMIS CATs. | O | O | O | O | O |  | Implementation |
| 55. | Management requires staff to use PROMIS CATs. | O | O | O | O | O |  | Implementation |
| 56. | Fellow therapists in my practice/institution use PROMIS CATs. | O | O | O | O | O |  | Implementation |
| 57. | In our practice/institution, we discuss how to implement PROMIS CATs. | O | O | O | O | O |  | Implementation |
| 58. | The use of PROMIS CATs fits well in the way of working in my practice/institution. | O | O | O | O | O |  | Acceptability |
| 59. | The use of PROMIS CATs is included in the policy of our practice/institution. | O | O | O | O | O |  | Acceptability |
| 60. | If the answer to question 59 is: completely disagree/disagree/not agree-disagree, then: What is the reason for this? | Free-text field | | | | |  |  |
|  |  | Completely agree | Agree | Don’t agree / don’t disagree | Disagree | Completely  disagree |  |  |
| 61. | I always have PROMIS CATs completed by the patients themselves. | O | O | O | O | O |  | Practicality |
| 62. | If the answer to question 61 is: completely disagree/disagree/not agree-disagree, then: For what reason did you or someone else help the patient to complete the PROMIS CAT? | Free-text field | | | | |  | Practicality |
| **Part 3. Inventory of the use of PROMIS CATs** | | | | | | | | |
| 63. | I use PROMIS CATs in .. % of my patients with musculoskeletal complaints | 0 0%  0 1-10%  0 11-20%  0 21-50%  0 51-80%  0 81-100% | | | | |  | Implementation |
| 64. | What are facilitators for the use of PROMIS CATs? | Free-text field | | | | |  | Implementation |
| 65. | What are barriers to the use of PROMIS CATs? | Free-text field | | | | |  | Implementation |
| **Part 4. Personal information** | | | | | | | | |
| 66. | Age (in years). |  | | | | |  |  |
| 67. | Gender. | 0 Male  0 Female  0 I don’t want to answer | | | | |  |  |
| 68. | Work experience as a physical therapist/exercise therapist/occupational therapist (in years): |  | | | | |  |  |
| 69. | Do you work in more than one practice? | 0 Yes  0 No | | | | |  |  |
| 70. | How many hours per week do you work as a physical therapist/exercise therapist/occupational therapist? |  | | | | |  |  |
| 71. | Specialization as (physical) therapist: | 0 Pediatric  0 Manual  0 Pelvic  0 Edema  0 Geriatric  0 Psychosomatic  0 Sports  0 Orofacial  0 Hand therapist  0 None | | | | |  |  |
| 72. | What kind of employment contract do you have? | 0 Employee  0 Practice owner  0 Self-employed without staff | | | | |  |  |
| **Part 5. Information about your practice** | | | | | | |  |  |
| 73. | Zip code of the practice (main location). |  | | | | |  |  |
| 74. | Location of the practice (main location). |  | | | | |  |  |
| 75. | Does your practice have more than one location? | 0 Yes  0 No | | | | |  |  |
| 76. | Number of physical therapists working in your practice (all locations combined). |  | | | | |  |  |
| 77. | Total number of full-time-equivalent (FTE) employees working in your practice (all locations combined). |  | | | | |  |  |
| 78. | Is there (a) practice assistant(s) working in your practice (all locations combined)? | 0 Yes  0 No | | | | |  |  |
| 79. | Is yes, for how many hours per week? |  | | | | |  |  |
| 80. | Is your practice affiliated with Fysiotopics? | 0 Yes  0 No | | | | |  |  |
| 81. | Is your practice affiliated with Zorg1? | 0 Yes  0 No | | | | |  |  |
|  | This is the end of the survey. Thank you very much for completing it! | | | | | |  |  |
