# Supplementary material for: Feasibility of patient-reported outcomes measurement information system (PROMIS) computerized adaptive tests in patients receiving physical therapy: A mixed-methods study
Source: PLoS One. 2026 Nov 13;21(11):e0362652. doi: 10.1371/journal.pone.0362652 (PMC13766268; doi:10.1371/journal.pone.0362652)
Supplement: Supplementary file 4 [file pone.0362652.s004.docx]

**Final interview guide for patients and physical therapists.**

| Introduction. Thank participant for conducting the interview. Start audio recording. Gain informed consent to audio recording. Demonstrate the PROMIS CAT and graphical feedback to refresh participants memory about the CAT | | |
| --- | --- | --- |
| **Main topic** | **Questions for patients** | **Questions for physical therapists** |
| Opening | What was your experience with PROMs in general when consulting the physical therapist? |  |
| CAT vs legacy PROMs | What was your experience with the PROMIS CAT (adaptive questionnaire)? | What was your experience with the PROMIS CAT (adaptive questionnaire)?  How do you see using PROMIS CATs in relation to the currently used (legacy) PROMs? |
| Completing during consultation or online | Where did you complete the CAT, online or during the consultation?  If online, what was your experience?  If during the consultation, what was your experience? | What were your experiences with completing the CAT (together with the patient) during the consultation?  What were experiences of your patients that completed the CAT online? |
| Time | What do you think about the time needed to complete the CAT? | What do you think about the time needed to use the CAT? |
| Usability and satisfaction |  | How easy was it for you to use PROMIS CAT?  How easy did you think your patients find it to use PROMIS CAT?  How satisfied were you with the CAT scores and graphical feedback? (e.g. heatmap, T-score, cut off values, no individual item scores visible) |
| Preparation to consult/influence on treatment plan | Did you have the idea that you were better prepared for the consultation after completing the CAT? | Can you tell us more about how using the PROMIS CAT influenced your physical therapy treatment plan? |
| Discussing outcomes | Did the physical therapist discuss the results /the CAT scores with you? If not, would you have liked so? | Can you tell us more about how you used the PROMIS CAT scores/graphical feedback within your physical therapy treatment plan.  Did you discuss scores, if yes when, if not why? |
| Aim | According to you, what is the aim and added value of using a CAT? |  |
| Implementation | What would be barriers or facilitators to implement this new type of PROM into physical therapy practice? | What would be barriers or facilitators to implement this new type of PROM into physical therapy practice?  What do you think about costs for completing CATs (12 -24 eurocents per CAT)  What other domains (other than Physical Function or Pain Interference) would you regard useful for primary care physical therapy?  Could using PROMIS CAT help you in communication with other healthcare professionals? |
| Knowledge and skills | What kind of knowledge or skills do you think a patient needs to complete a CAT? | What kind of knowledge or skills do you think you need to use PROMIS CAT (e.g. interpretation of results)? |
|  | Collecting of descriptive data. Thank participant and finish interview | |
