# Supplementary material for: Feasibility of patient-reported outcomes measurement information system (PROMIS) computerized adaptive tests in patients receiving physical therapy: A mixed-methods study
Source: PLoS One. 2026 Nov 13;21(11):e0362652. doi: 10.1371/journal.pone.0362652 (PMC13766268; doi:10.1371/journal.pone.0362652)
Supplement: Supplementary file 5 [file pone.0362652.s006.docx]

Response to Reviewers

PONE-D-26-14648

Feasibility of Patient-Reported Outcomes Measurement Information System (PROMIS®) Computerized Adaptive Tests in patients receiving physical therapy: A mixed-methods study

Dear Academic Editor and Reviewer,

Thank you for your careful review of our manuscript. Below, we provide a point-by-point response to the journal requirements and to each comment raised by the Academic Editor and Reviewer. All corresponding changes have been incorporated into the revised manuscript.

**Journal requirements:**

1. Please ensure that your manuscript meets PLOS ONE's style requirements, including those for file naming.

**Response:** We carefully rechecked the manuscript against the PLOS ONE style requirements and made the following changes:

- We updated the author affiliations, to reflect the current institutional affiliations. Amsterdam Public Health (APH) is listed as a separate affiliation, and Dr Terwee’s affiliation with APH Quality of Care has been added. The affiliation of Dr. Kiers with the Department of Human Movement Sciences, Vrije Universiteit Amsterdam, has been removed, as he is not currently affiliated with this department.
- We added the initials of the corresponding author’s (EH and HK) in parentheses after their respective email addresses.
- We removed the running title “Feasibility of PROMIS CATs in patients receiving physical therapy”.
- We adjusted the formatting of Level1, 2 and 3 headings to bold type, using font sizes of 18, 16 and 14 pt, respectively.
- We standardized the initials of authors and other contributors throughout the manuscript by removing the periods (e.g., EH instead of E.H.).
- We formatted all paragraphs in the main body of the manuscript with a first-line indent, in accordance with the PLOS ONE Manuscript Body Formatting Guidelines.
- We checked the manuscript file names for compliance with the journal’s file naming requirements.

2. We note that the grant information you provided in the ‘Funding Information’ and ‘Financial Disclosure’ sections do not match. When you resubmit, please ensure that you provide the correct grant numbers for the awards you received for your study in the ‘Funding Information’ section.

**Response:** Thank you for noting the discrepancy between the Funding Information and Financial Disclosure sections. We have reviewed and corrected the funding information to ensure that all funding sources, grant numbers, funding recipients, and funder details are reported consistently.

Funding from the Netherlands Organisation for Health Research and Development (ZonMw), which contributed to the project, was inadvertently omitted from the original submission and has now been added. The complete corrected Financial Disclosure and Role of Funders statement are provided in the Funding Information and Financial Disclosure sections.

3. Financial disclosure. Please state wat the role the funders took in the study.

**Response:** The funders Taskforce for Applied Research SIA RAAK, part of the Dutch Research Council (NWO) and ZonMW (The Netherlands Organisation for Health Research and Development) had no role. We added the sentence “These funders had no role in study design, data collection and analysis, decision to publish, or preparation of the manuscript” in the Role of Funder statement. We clarified the financial support for the use of PROMIS CATs, provided by the Association for Quality in Physical Therapy (Stichting Keurmerk Fysiotherapie [SKF]). The role of H. Kiers, whose position as chair of SKF was already disclosed in the original submission, has been further clarified to include his involvement in the funding decision.

4. Competing Interest Section. (..) Please confirm that this does not alter your adherence to all PLOS ONE policies on sharing data and materials, by including the following statement: "This does not alter our adherence to PLOS ONE policies on sharing data and materials.”

**Response:** We clarified the role of co-author H.Kiers as a chair of SKF and his involvement in the decision by SKF to provide financial support for the use of PROMIS CATs. We also added the sentence “This does not alter our adherence to PLOS ONE policies on sharing data and materials” in the Competing Interests section.

5. We note that you have indicated that there are restrictions to data sharing for this study.

**Response:** Thank you for this comment. The data cannot be shared publicly because the qualitative interview data contain potentially identifying or sensitive information and cannot be completely anonymized. Public sharing would therefore not be consistent with the conditions under which the data were collected and approved by the Ethical Committee of Research in Health of HU University of Applied Sciences Utrecht. The data are available from the ResearchDrive at HU University of Applied Sciences Utrecht upon reasonable request by researchers who meet the criteria for access to confidential data. Data requests can be directed to [onderzoeksupport@hu.nl](mailto:onderzoeksupport@hu.nl).

The Data Availability Statement in the submission has been reviewed to ensure that this information is clearly stated.

6. If the reviewer comments include a recommendation to cite specific previously published works, please review and evaluate these publications to determine whether they are relevant and should be cited. There is no requirement to cite these works unless the editor has indicated otherwise.

**Response:** Thank you for this clarification. The reviewer did not recommend citing any specific previously published works; therefore, no changes were required in response to this point.

7. Reference list. Please review your reference list to ensure that it is complete and correct.

**Response:** We carefully reviewed the reference list and confirmed that it is complete and correct.

Additional Editor Comments:

Thank you for your additional comments and for noting that this is a practical and well-written paper. Below, we respond to each of your suggestions point by point.

1. The following interpretations may conflict with the quantitative results. The narrative should be carefully audited against the tables. Examples include:

   Lines 365–367 state that “nearly all” therapists rated CATs as easy to use, but only 72% agreed or completely agreed.
   Lines 412–413 state that “few” therapists felt confident interpreting CAT scores, while 68% agreed that they could correctly interpret them and 88% reported knowing how to apply them within clinical reasoning.

**Response:** Thank you for this comment. We reviewed the narrative and the tables and made the following changes:

Line 277-278 “CAT results were not always discussed with patients: 48% reported doing so always or mostly.”, we added “(Table 3a)”.

Line 328 “Most PTs expressed interest in using PROMIS CATs more routinely (80%)…”, we deleted “more routinely” as this was not exactly stated as such in the question.

Line 330 “More than half wished to increase their usage.”, we added (56%).

Lines 365-367: “nearly all” was changed into “most” and we added “(72%).

Lines 411-412 We changed “Few” therapists into “In general, PTs” and in line 412 we added “However, ..”, to stress the discrepancy between these findings.

2. Table 2 requires a complete audit.

**Response:** Thank you for identifying these inconsistencies. We conducted a complete audit of Table 2 and corrected the counts and percentages where necessary.

For item 15, an error occurred when transferring the data to the table. The counts and corresponding percentages have been corrected so that they are based on the total of 25 respondents and sum to 100%.

For item 22, the percentage in the “% agreeing” column has been corrected from 38% to 48% (12/25).

For items 42 and 43, three respondents selected “not applicable/did not use”, resulting in an applicable denominator of n = 22. For item 44, one respondent selected “not applicable/did not use”, resulting in an applicable denominator of n = 24. Although these denominators were correctly stated in the table footnotes, the percentages displayed in the original table had inadvertently been calculated using the total sample of 25. We have recalculated all percentages for these items using the applicable denominators. The percentages in the “% agreeing” column have also been corrected accordingly.

The revised percentages are 50.0% for item 42, 45.5% for item 43, and 58.3% for item 44. The table footnotes have been retained to clarify the applicable denominators.

3. Whether coding disagreements were documented and how consensus was reached.

**Response:** Thank you for this comment. We agree that the procedure for resolving coding disagreements was described too briefly. We have expanded the qualitative analysis section to clarify the process. The two researchers who independently coded each initial transcript compared their codes during consensus meetings and resolved differences through discussion. When they could not reach consensus, H.W. reviewed the relevant transcript excerpt and codes and made the final decision. Coding disagreements were documented in a separate disagreement log; the resulting agreed codes were incorporated into the coding framework in ATLAS.ti.

The revised manuscript now states: “Two researchers (E.H. and A.J. or A.D.) independently performed open coding of each initial transcript. The two coders compared their codes during consensus meetings and resolved differences through discussion. When consensus could not be reached, H.W. reviewed the relevant transcript excerpt and codes and made the final decision. Coding disagreements were documented in a separate disagreement log.”

4. Please provide more information on when and how quantitative and qualitative findings were integrated and how agreement and disagreement between quantitative and qualitative were evaluated.

**Response:** Thank you for this comment. We agree that the timing and process of mixed-methods integration were insufficiently described. We have added a separate paragraph to the Methods section explaining that the quantitative and qualitative data were initially analyzed separately and were subsequently integrated during the interpretation phase.

The first and second authors compared the survey results and interview themes within each of Bowen’s four feasibility domains. Findings were evaluated for convergence, complementarity, and divergence. Convergence was identified when both data sources pointed in the same direction; complementarity when the qualitative findings provided additional explanation or context for the quantitative results; and divergence when findings appeared inconsistent or reflected contrasting experiences. Differences were discussed while considering that the survey included PTs only, whereas the interviews included both PTs and patients. No formal concordance score was used. The results of this comparison were presented as an adapted and broadened integrated summary for each feasibility domain.

The following paragraph has been added to the Methods section: “Quantitative and qualitative data were initially analyzed separately. Following completion of both analyses, the first and second author (E.H. and A.D.) compared the survey results and interview themes within each of Bowen’s feasibility domains: acceptability, demand, practicality, and implementation. Findings were considered convergent when the quantitative and qualitative results pointed in the same direction, complementary when the interview findings provided further explanation or context for the survey results, and divergent when findings appeared inconsistent or reflected contrasting experiences. Potential differences were discussed, taking into account that the survey included PTs only, whereas the interviews included both PTs and patients. Agreement and disagreement were evaluated qualitatively rather than by using a formal concordance score. This comparison informed the integrated summary presented for each feasibility domain in the Results section.”

**Reviewer’s Reponses to Questions**

Thank you for reviewing our manuscript.

Response: Thank you for your positive reaction to Questions 1 to 4.

5. **Comments to the Author**

Reviewer #1: line 140 - “insufficient command of the Dutch language” - how was insufficient proficiency defined or assessed, and how was it presented in the questionnaire?

**Response:** Thank you for requesting clarification. Insufficient command of the Dutch language was not assessed using a standardized language proficiency test or predefined cut-off, nor was it included as an item in the questionnaire for physical therapists. The assessment was based on the physical therapist’s clinical judgement of whether the patient could sufficiently understand the Dutch-language study information and questionnaires to participate.

We have clarified this procedure in the Methods section as follows: “Based on clinical judgement, the treating PT assessed whether the patient could sufficiently understand the Dutch-language study information and questionnaires to participate. No standardized language proficiency assessment or predefined cut-off was used. This criterion was not included as an item in the questionnaire for PTs.”

line 149 - Please clarify the phrase “beginning with an item near the scale mean”. What specific value does the “scale mean” refer to? Does this indicate an item with a difficulty parameter near the mean of the item bank?

**Response:** Thank you for requesting clarification. The phrase “the mean of the item bank scale” refers to the center of the latent trait scale, θ = 0, which corresponds to a PROMIS T-score of 50. Thus, the first item is calibrated to target the middle of the item bank scale. This does not refer to the arithmetic mean of the item difficulty parameters across all items in the bank. The response to this first item is used to obtain the initial T-score estimate, after which the most informative subsequent items are selected iteratively based on the updated estimate.

We have clarified the manuscript as follows: “Each DF-PROMIS CAT begins with the most informative item for the average of the general population and the patient’s response is used to estimate a T-score and its corresponding standard error.”

line 151 - Would there a be reference or justification for using a standard error stopping rule of 2.24 for PROMIS CATs? It would be helpful to explain why this threshold was selected.

**Response:** Thank you for requesting justification of the stopping rule. At the time of this study, the Dutch-Flemish PROMIS CATs used an SE(T-score) threshold of 2.24. This threshold corresponds to a reliability of approximately 0.95 and had historically been selected for clinical use in the Netherlands to obtain highly precise scores at the individual-patient level. The same stopping rule and its relationship with score reliability is described by Arensman et al. [14].

We have added this rationale to the Methods section. Since this study was conducted, the Dutch-Flemish PROMIS National Center has adopted a less stringent stopping rule of SE(T-score) < 3.16, corresponding to a reliability of approximately 0.90, to align more closely with international PROMIS practice and to limit respondent burden when multiple domains are administered.

line 155 - the manuscript states that “Each domain was displayed in separate graph” - Including an example of this graphical display would be helpful for readers.

**Response:** Thank you for this helpful suggestion. We agree that an example of the graphical display could assist readers. However, the graphs were generated and displayed within the EHR system and could not be exported directly as publication-quality image files. Only screen captures were available, which did not provide sufficient quality to meet the journal’s technical figure requirements. We therefore decided not to add a separate figure.

To improve clarity, we have expanded the description of the graphical feedback in the Methods section and now explicitly direct readers to reference [25], which provides visual examples and a detailed explanation of this graphical display.

The revised manuscript states: “Each domain was displayed in a separate longitudinal graph, in which numerical T-scores were presented as dots connected by a blue line. The graph was overlaid on a traffic-light heat map indicating reference cut-off ranges, with orange representing moderate and red representing severe scores. A visual example and further explanation of this graphical display are provided by Van Muilekom et al. [25].”

line 192 - “Guides were piloted in three test interviews to improve question clarity and ensure clear distinction between PROMIS CATs and legacy PROMs” - Please provide additional information about this pilot processes. For example, were there any major revisions or improvements made after each pilot process?

**Response:** Thank you for requesting further information about the pilot process. The three test interviews were used both to refine the topic guides and to train and align the interviewers. No major revisions were made to the overall content or the feasibility domains after the individual pilot interviews. However, several minor but relevant improvements were made to the wording, sequence, and administration of the interviews.

The interviews were revised to begin with the participant’s presenting complaint and the questionnaires they had completed. To ensure a clear distinction between PROMIS CATs and legacy PROMs throughout the interview, interviewers were instructed to show an example of a PROMIS CAT, including its graphical feedback, alongside an example of a legacy PROM. Prompts were added about whether CAT completion occurred at home or during the consultation and whether participants wished to view individual item responses. Questions concerning usability and implementation were more clearly separated.

The pilot interviews were also used to train interviewers to ask open and non-leading questions, use follow-up probes and summaries, allow pauses, and conduct the interview as a conversation rather than follow the guide as a fixed list of questions. We have added this information to the Methods section (lines 235 – 245).

lines 219-222 Please replace decimal commas with decimal points(.)

**Response:** Thank you for noting this. We have replaced the decimal commas with decimal points in the Results section and reviewed the remainder of the manuscript and tables for consistent decimal notation.

line 220 - Should be nine patients, instead of 9 patients, consistent with the previous sentences.

**Response:** Thank you. We have replaced “9 patients” with “nine patients” for consistency with the surrounding text.

line 221 - This sentence is difficult to follow because it combines characteristics of PTs and patients. It could be divided into two sentences or consider revising it as follows: Interview participants varied in age, years of experience, clinical focus among PTs, symptom location, education levels, and digital skills among patients.

**Response:** Thank you for this suggestion. We agree that separating the characteristics of PTs and patients improves readability. We have divided the original sentence into two sentences as suggested (lines 300-302). For consistency, we changed “Technological skills” into “Digital skills” in Table 1b, so that it is in line with the text.

Lines 225 - 231
- Was there a predefined criteria for selecting 10 PTs out of 25 who completed the survey?

**Response:** Thank you for requesting clarification. We aimed a priori to conduct approximately ten interviews with PTs and ten with patients. This was a pragmatic target that the research team considered sufficient to obtain data richness and thematic coverage within the scope of this mixed-methods feasibility study. We did not use a formal saturation threshold.

There were no additional predefined criteria for selecting ten specific PTs from the PTs who completed the survey. PTs were recruited by convenience sampling based on their willingness and availability to participate. Although purposive sampling was initially planned to obtain variation in participant characteristics, limited willingness to participate, particularly among patients, resulted in reliance on convenience sampling. We therefore interviewed ten PTs and nine patients.

We have clarified the target sample size and recruitment procedure in the Methods section. The Methods section now clarifies that purposive sampling was initially planned, but that no formal selection criteria or quotas were predefined and that recruitment ultimately relied on convenience sampling.

Table 1a - Revise title to: Characteristics “of” interviewed “physical” therapists
Table 1b - Revise title to: Characteristics “of” interviewed patients

**Response:** thank you for noting. We made changes as suggested.

In Table 1b, education level (High, Middle, Low) and technological skills (High, Average) - please explain how were they defined and classified.

**Response:** Thank you for requesting clarification. Education level and digital skills were self-reported by participants using pragmatic categorical response options. Education level was reported as high, middle, or low, and digital skills as high, average, or low. No standardized definitions or classification instruments were used. We have clarified this in the Methods section and changed the headings in Table 1b to “Self-reported education level” and “Self-reported digital skills.”


Line 510 - the manuscript mentions that some cases for UE CAT reached the max 12 items, but could not be confirmed. If available, it would be informative to report the mean, standard deviation, and range of the number of items administered for each CAT, and compare these values with that of the legacy PROMs. This comparison would quantify and demonstrate the relative efficiency of PROMIS CATs. Please also consider reporting the mean, standard deviation, and range of the resulting T-scores for PROMIS CATs.

**Response:** Thank you for this suggestion. We agree that information about the number of administered items could provide an objective indication of CAT efficiency. However, the number of items administered during individual CAT assessments was not available in the dataset used for this feasibility study. We therefore could not calculate the mean, standard deviation, or range of the number of items per CAT or compare these values with the number of items completed for the legacy PROMs. In addition, the legacy PROMs differed between patients, and some were administered in combination, which would make a direct comparison of item counts difficult to interpret.

We have revised the Discussion to clarify that reports of the UE CAT being relatively long were participants’ perceptions and could not be verified using objective CAT administration data. The original suggestion that some CATs may have reached the maximum of twelve items has therefore been removed.

We also considered adding descriptive statistics for the PROMIS T-scores. However, the primary aim of the present study was to evaluate the feasibility and experiences of using PROMIS CATs in routine physical therapy rather than to describe the distribution of clinical outcome scores. Descriptive and psychometric results for the PROMIS CAT scores in this cohort have been reported by Arensman et al. [14, 15], whose studies are already cited in the Introduction and Methods sections. We therefore did not duplicate these analyses in the present manuscript.

Line 527 - the manuscript mentions “14 practices, 25 PTs” here, whereas it was spelled out as “twenty-five” earlier in the Results section. Please ensure consistent formatting of numbers throughout the manuscript.

**Response:** Thank you for noting this inconsistency. We have reviewed the entire manuscript and standardized the presentation of numbers. Numbers from one to nine are now spelled out in running text, whereas numbers of 10 or higher are presented as numerals, except when a number occurs at the beginning of a sentence. Numerals are also used consistently for percentages, measurements, dates, durations, and statistical results.

Line 145 on PROMIS CAT integration - the manuscript mentions that “CAT administration occurred alongside the use of legacy PROMs as part of routine care”. Please clarify the order in which the PROMIS CATs and legacy PROMs were administered. Was the order standardized, or did it vary across participants or clinical settings?

**Response:** Thank you for this comment. The order of administration was not standardized because each practice integrated the PROMIS CATs into its own electronic health record–based data collection workflow. We therefore did not control the order in which PROMIS CATs and legacy PROMs were administered, and this may have varied across practices and participants. We have clarified this in the Methods section (lines 208–211).

Line 560 - The phrase “which scores” is grammatically unclear. Please consider restructuring the sentence. In addition, “aggravated level” appears to be a typo for “aggregated level”.

**Response:** Thank you for identifying these issues. We have restructured the sentence for clarity and corrected “aggravated level” to “aggregated level.” The revised sentence now reads: “Such an approach could preserve the efficiency and comprehensiveness of PROMIS CATs and allow their scores to be used for quality evaluation at an aggregated level, while supporting the individualized goal-setting processes that PTs consider essential in daily practice.”

- End of document -
